# XRCC1 protects transcription from toxic PARP1 activity during DNA base excision repair

**DOI:** 10.1038/s41556-021-00792-w

**Published:** 2021-11-22

**Authors:** Marek Adamowicz, Richard Hailstone, Annie A. Demin, Emilia Komulainen, Hana Hanzlikova, Jan Brazina, Amit Gautam, Sophie E. Wells, Keith W. Caldecott

**Affiliations:** 1 Genome Damage and Stability Centre and Sussex Drug Discovery Centre, School of Life Sciences, University of Sussex, Brighton, UK; 2 Department of Genome Dynamics, Institute of Molecular Genetics of the Czech Academy of Science, Prague, Czech Republic

**Keywords:** Single-strand DNA breaks, Transcription, Histone post-translational modifications, Ubiquitylation

## Abstract

Genetic defects in the repair of DNA single-strand breaks (SSBs) can result in neurological disease triggered by toxic activity of the single-strand-break sensor protein PARP1. However, the mechanism(s) by which this toxic PARP1 activity triggers cellular dysfunction are unclear. Here we show that human cells lacking XRCC1 fail to rapidly recover transcription following DNA base damage, a phenotype also observed in patient-derived fibroblasts with XRCC1 mutations and *Xrcc1*^−/−^ mouse neurons. This defect is caused by excessive/aberrant PARP1 activity during DNA base excision repair, resulting from the loss of PARP1 regulation by XRCC1. We show that aberrant PARP1 activity suppresses transcriptional recovery during base excision repair by promoting excessive recruitment and activity of the ubiquitin protease USP3, which as a result reduces the level of monoubiquitinated histones important for normal transcriptional regulation. Importantly, inhibition and/or deletion of PARP1 or USP3 restores transcriptional recovery in *XRCC1*^−/−^ cells, highlighting PARP1 and USP3 as possible therapeutic targets in neurological disease.

## Abstract (web summary)

Adamowicz et al. report that toxic PARP1 activity, induced by ataxia-associated mutations in XRCC1, impairs the recovery of global transcription during DNA base excision repair by promoting aberrant recruitment and activity of the histone ubiquitin protease USP3.

## Main

DNA single-strand breaks (SSBs) are one of the most common types of DNA damage in cells, arising at a frequency of tens-of-thousands per cell per day^1^. The threat posed by SSBs is illustrated by hereditary genetic diseases in which the repair of SSBs is defective, resulting in neurological pathologies such as cerebellar ataxia, neurodevelopmental delay and seizures^2,3^. Single-strand breaks are rapidly detected by poly-ADP-ribose polymerase 1 (PARP1), which following binding to the DNA break is catalytically activated and modifies itself and other proteins, such as histones, with ADP-ribose^4–6^. ADP-ribosylation accelerates SSB repair in a number of ways—for example, by modifying the structure of chromatin in the vicinity of the break and/or recruiting specific DNA repair factors such as XRCC1 (refs. ^4,6^). The extensive auto-ribosylation of PARP1 leads to disassociation of the enzyme from the SSB, enabling other DNA repair enzymes to access and repair the break^7^.

XRCC1 is a scaffold protein that is recruited to SSBs by PARP1 and/or PARP2 activity through direct interaction between poly(ADP-ribose) and the central BRCT domain in XRCC1 (refs. ^8–10^). XRCC1 in turn interacts with and recruits the enzymes required for SSB repair, including DNA polymerase β^11,12^, DNA ligase III^13^, polynucleotide kinase/phosphatase^14,15^ and aprataxin^16,17^. Hereditary mutations in *XRCC1* and some of these protein partners result in neurodevelopmental disorders and/or progressive neurodegeneration^2,3^. Intriguingly, the deletion and/or inhibition of PARP1 greatly reduces or prevents these pathologies in an *Xrcc1*-defective mouse model of SSB-associated neurological disease, highlighting excessive/aberrant PARP1 activity as a source of SSB-induced neuropathology^18,19^. However, the molecular mechanism by which excessive/aberrant PARP1 activity triggers cellular dysfunction remains unclear.

Here we show that prolonged PARP1 activity at unrepaired DNA base damage suppresses the recovery of transcription by promoting the excessive recruitment and activity of the ubiquitin-specific protease USP3. Remarkably, excessive USP3 activity reduces global levels of monoubiquitinated histones important for transcriptional regulation, such as histone H2A and H2B monoubiquitination at K119 and K120 (H2AmUb and H2BmUb, respectively). Importantly, either PARP1 or USP3 inhibition/depletion rescue normal levels of histone monoubiquitination and transcription recovery following DNA base damage, highlighting these enzymes as possible therapeutic targets in the treatment of base excision repair (BER)-defective neurological disease.

## Results

### XRCC1 promotes the recovery of transcription following oxidative damage

**Fig. 1:**
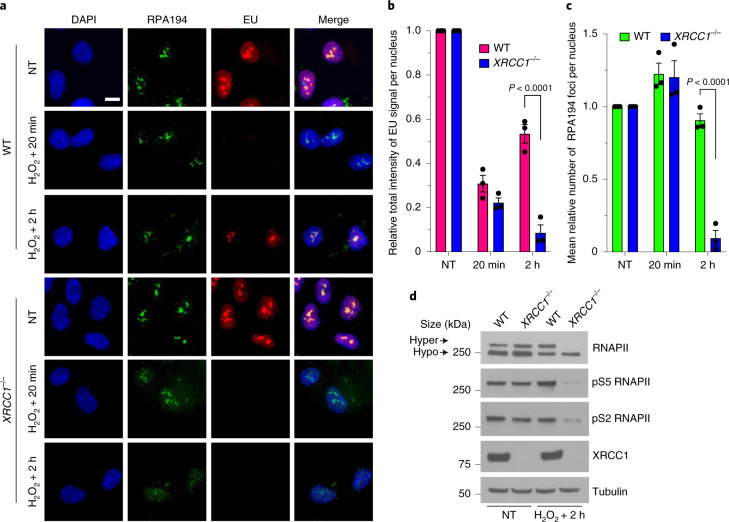
XRCC1 promotes the recovery of transcription following oxidative damage. **a**, Representative images of RNAPI foci (RPA194) showing the levels of global transcription (EU pulse labelling) in WT and *XRCC1*^−/−^ RPE-1 cells following mock treatment (NT) or at the indicated times after treatment with 250 μM H_2_O_2_ for 5 min. The cells were labelled with EU for 20 min before fixation. Scale bar, 10 μm; DAPI, 4,6-diamidino-2-phenylindole. **b**,**c**, Levels of global transcription (**b**; EU immunofluorescence) and RNAPI foci (**c**; RPA194) from the experiment shown in **a**. Data are the mean ± s.e.m. of three independent experiments. Statistical significance was determined using a two-way analysis of variance (ANOVA) with Sidak’s multiple comparisons test (significantly different *P* values are indicated). **d**, Immunoblot of RNAPII hyperphosphorylation (hyper) in WT and *XRCC1*^−/−^ RPE-1 cells following mock treatment or 2 h after treatment with 250 μM H_2_O_2_ for 5 min. A representative blot from one of three independent experiments is shown. The positions of the molecular mass markers are indicated; hypo, hypophosphorylation. Source data

**Extended Data Fig. 1:**
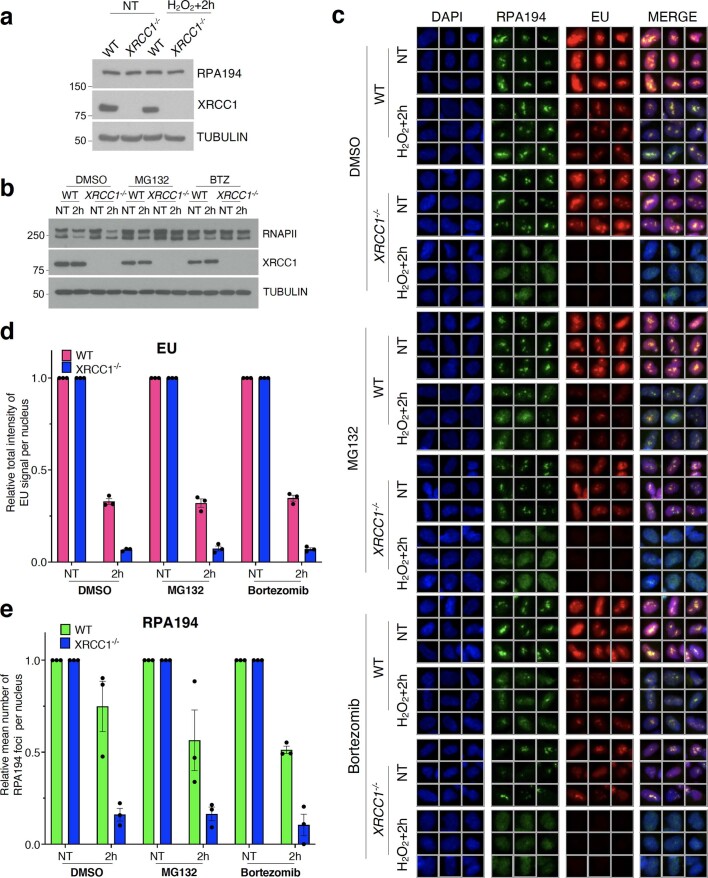
XRCC1 promotes the recovery of transcription following oxidative damage. **a**, Immunoblot of RPA194 in WT and *XRCC1*^*−/−*^ RPE-1 cells following mock treatment or 2 h after treatment with 250 μM H_2_O_2_ for 5 min. A representative image from one of three independent experiments is shown. **b**, Immunoblot of RNAPII hyperphosphorylation in WT and *XRCC1*^*−/−*^ RPE-1 cells following mock treatment or 2 h after treatment with 250 μM H_2_O_2_ for 5 min. Where indicated, cells were pretreated with either 10 μM MG132 or 1 μM Bortezomib for 1 h before H_2_O_2_ treatment. A representative blot from one of three independent experiments is shown. **c**, RNAPI foci (RPA194) and levels of global transcription (EU immunofluorescence) in WT and *XRCC1*^*−/−*^ RPE-1 cells pretreated for 1 h with 10μM MG132 or 1 μM Bortezomib following mock treatment or at 2 h after treatment with 250 μM H_2_O_2_ for 5 min. **d** and **e**, Quantification of the levels of global transcription (EU immunofluorescence) and RNAPI foci (RPA194) shown in **(c)**. Data are means (±s.e.m.) of three independent experiments. Source data

Given that SSBs can slow or block the physical progression of RNA polymerases^20–22^, we wondered whether SSB repair-defective cells exhibit defects in transcription following DNA damage. We therefore examined the impact of XRCC1 deletion on global transcription in human RPE-1 cells following treatment with H_2_O_2_ (a physiologically relevant source of oxidative SSBs) by pulse labelling with 5-ethynyluridine (EU). We observed a robust inhibition of global transcription immediately after treatment with H_2_O_2_ in both wild-type (WT) and *XRCC1*^−/−^ RPE-1 cells (Fig. 1a,b). More importantly, whereas the levels of transcription recovered significantly within 2 h following H_2_O_2_ treatment in the WT RPE-1 cells, they declined further in the *XRCC1*^−/−^ cells (Fig. 1a,b). The lack of transcription recovery in *XRCC1*^−/−^ cells was accompanied by a progressive loss of RNA polymerase I (RNAPI) foci (RPA194; Fig. 1a,c). Given that we did not detect a reduction in the global levels of RPA194 in the *XRCC1*^−/−^ cells, this most probably reflected the dissociation of RNAPI from nucleolar sites of ribosomal RNA transcription rather than degradation of the RNAP (Extended Data Fig. 1a).

**Extended Data Fig. 2:**
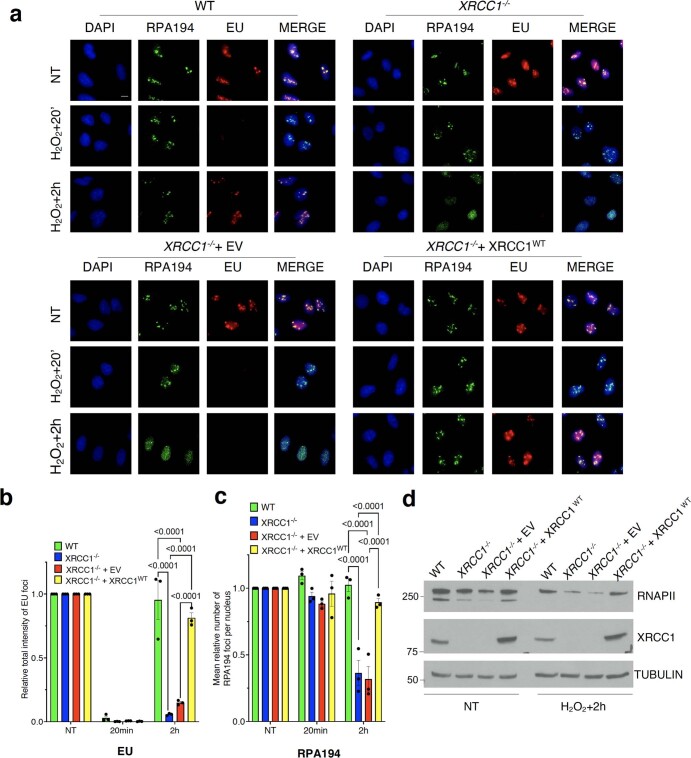
XRCC1 promotes the recovery of transcription following oxidative damage. **a**, Representative images of the RNAPI foci (RPA194) and levels of global transcription (EU immunofluorescence) in WT and *XRCC1*^*−/−*^ U2OS cells stably transfected with either empty vector (EV) or expression construct encoding full length C-terminal histidine-tagged XRCC1 (XRCC1^WT^), following mock treatment or at the indicated times after treatment with 1 mM H_2_O_2_ for 20 min. Scale bars, 10 μm. **b** and **c**, Quantification of the RNAPI foci (RPA194) and levels of global transcription (EU immunofluorescence) shown in **(a)**. Data are means (±s.e.m.) of three independent experiments, and statistically significant differences were determined by two-way ANOVA with Tukey’s multiple comparisons test (p values are indicated). **d**, Immunoblot of RNAPII hyperphosphorylation in WT U2OS cells, *XRCC1*^*−/−*^ U2OS cells, and *XRCC1*^*−/−*^ U2OS cells stably transfected with either empty vector (EV) or expression construct encoding full-length histidine-tagged XRCC1 (XRCC1^WT^), following mock treatment or 2 h after treatment with 1 mM H_2_O_2_ for 20 min. A representative blot from one of three independent experiments is shown. Source data

Although most of the EU signal detected in our pulse-labelling experiments was nucleolar, the recovery of EU incorporation in the *XRCC1*^−/−^ RPE-1 cells was also reduced in the nucleoplasm (Fig. 1a), suggesting that RNA polymerase II (RNAPII)-dependent transcription was also affected. To confirm this, we compared the levels of hyperphosphorylated RNAPII, which is an established measure of the RNAPII transcriptional activity^23,24^, in WT and *XRCC1*^−/−^ RPE-1 cells before and after H_2_O_2_ treatment. The level of RNAPII hyperphosphorylation was markedly reduced in the *XRCC1*^−/−^ RPE-1 cells following H_2_O_2_ treatment, an observation we verified with antibodies specific to two phosphorylated sites (S2 and S5) within the carboxy (C)-terminal domain of RNAPII that are associated with transcriptional activity (Fig. 1d)^25^. The loss of hyperphosphorylated RNAPII seemed to involve, at least partly, proteolysis of RNAPII, given that incubation with proteasome inhibitors restored normal levels of the hyperphosphorylated RNAP (Extended Data Fig. 1b). However, proteasome inhibitors did not restore normal levels of transcription recovery, indicating that degradation of hyperphosphorylated RNAPII was a result of failed transcription recovery rather than a cause (Extended Data Fig. 1b–e). Importantly, the defect in transcription recovery was neither limited to RPE-1 cells nor an off-target effect of gene editing because we observed similar results in *XRCC1*^−/−^ U2OS cells and the defect in transcriptional recovery in these cells was corrected by stable expression of recombinant human XRCC1 (Extended Data Fig. 2a–d). Collectively, these data indicate that XRCC1 is required for the rapid recovery of transcription by both RNAPI and RNAPII following oxidative stress.

### Reduced transcriptional recovery in XRCC1-deficient cells and neurons is a result of PARP1

**Fig. 2:**
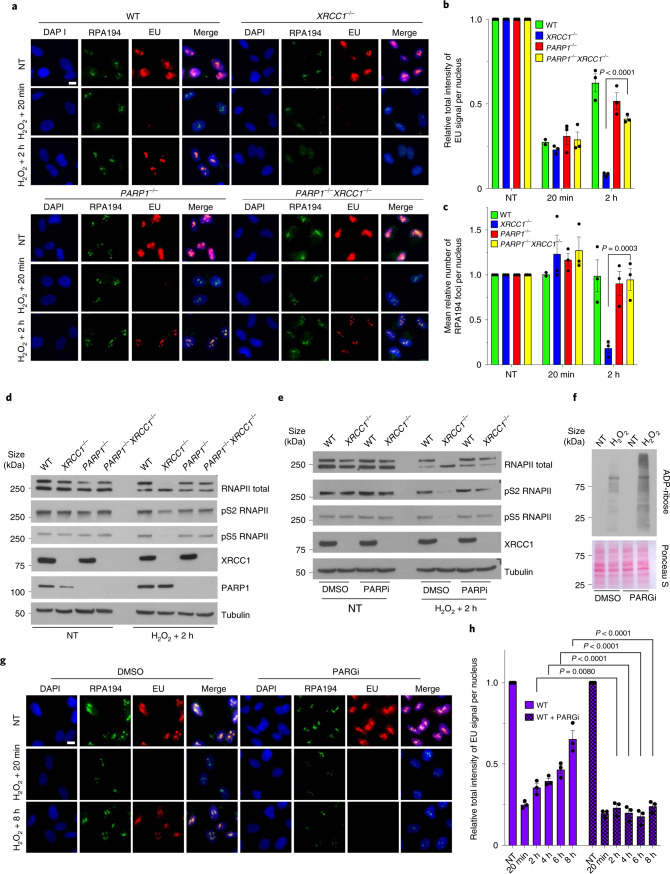
Reduced transcriptional recovery in XRCC1-deficient cells is a result of toxic PARP1 activity. **a**, Representative images of RNAPI foci (RPA194) showing the levels of global transcription (EU pulse labelling) in WT, *XRCC1*^−/−^, *PARP1*^−/−^ and *PARP1*^−/−^*XRCC1*^−/−^ RPE-1 cells following mock treatment or at the indicated times after treatment with 250 μM H_2_O_2_ for 5 min. The cells were incubated with dimethylsulfoxide (DMSO) vehicle or 10 μM PARPi for 1 h before, during and following H_2_O_2_ treatment as indicated. The cells were pulse labelled with EU as in Fig. 1. **b**,**c**, Levels of global transcription (**b**; EU immunofluorescence) and RNAPI foci (**c**; RPA194) from the experiment shown in **a**. **d**, Immunoblot of RNAPII hyperphosphorylation in WT, *XRCC1*^−/−^, *PARP1*^−/−^ and *PARP1*^−/−^*XRCC1*^−/−^ RPE-1 cells following mock treatment or 2 h after treatment with 250 μM H_2_O_2_ for 5 min. **e**, Immunoblot of RNAPII hyperphosphorylation in WT and *XRCC1*^−/−^ RPE-1 cells following mock treatment or 2 h after treatment with 250 μM H_2_O_2_ for 5 min. The cells were incubated with DMSO vehicle or 10 μM PARPi for 1 h before, during and following H_2_O_2_ treatment as indicated. **f**, Immunoblot of the poly(ADP-ribose) levels in WT RPE-1 cells treated with PARG inhibitor following mock treatment or treatment with 250 μM H_2_O_2_ for 5 min. Ponceau S staining is also shown as a loading control. For **d**–**f**, representative blots from one of three independent experiments are shown. **g**, Representative images of RNAPI foci (RPA194) and levels of global transcription (EU pulse labelling) in WT RPE-1 cells treated with PARG inhibitor following mock treatment or at the indicated times after treatment with 250 μM H_2_O_2_ for 5 min. **h**, Levels of global transcription (EU immunofluorescence) from the experiment shown in **g**. **b**,**c**,**h**, Data are the mean ± s.e.m. of three independent experiments. Statistical significance was determined using a two-way ANOVA with Tukey’s (**b**,**c**) or Sidak’s (**h**) multiple comparisons test (significantly different *P* values are indicated). Scale bars, 10 μm. PARGi, PARG inhibitor. Source data

**Fig. 3:**
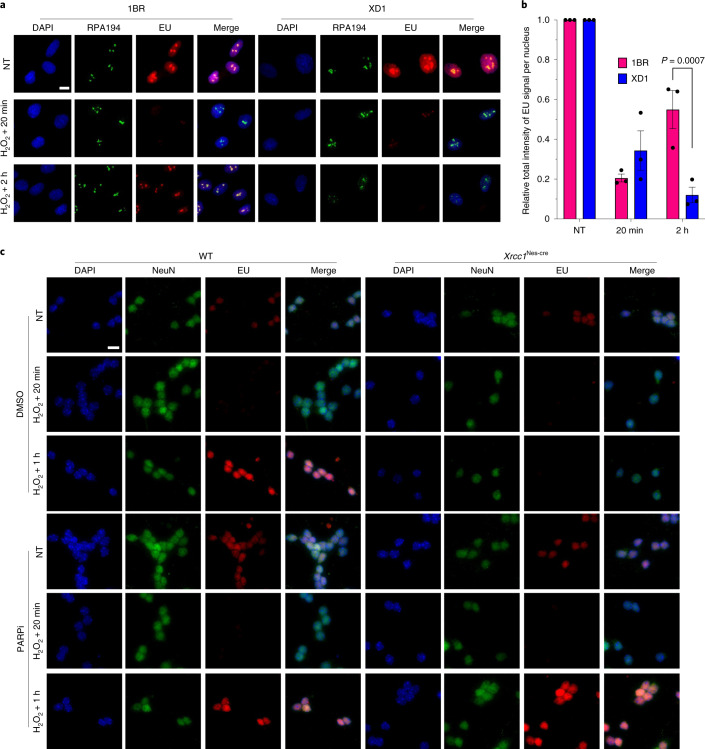
Toxic PARP1 activity suppresses transcriptional recovery in patient-derived fibroblasts with XRCC1 mutations and Xrcc1-deficient mouse cerebellar neurons. **a**, Representative images of RNAPI foci (RPA194) showing the levels of global transcription (EU pulse labelling) in normal (1BR) and patient-derived fibroblasts with XRCC1 mutations (XD1) following mock treatment or at the indicated times after treatment with 60 μM H_2_O_2_ for 5 min. The cells were pulse labelled with EU as in Fig. 1. **b**, Levels of global transcription (EU immunofluorescence) from the experiment shown in **a**. Data are the mean ± s.e.m. of three independent experiments. Statistical significance was determined using a two-way ANOVA with Sidak’s multiple comparisons test (significantly different *P* values are indicated). **c**, Representative images of the global transcription levels (EU immunofluorescence) in WT and *Xrcc1*^Nes-cre^ mouse cerebellar neurons (NeuN), pretreated with PARPi, following mock treatment or at the indicated times after treatment with 250 μM H_2_O_2_ for 5 min. Representative images from one of three independent experiments are shown. Scale bars, 10 μm. Source data

**Extended Data Fig. 3:**
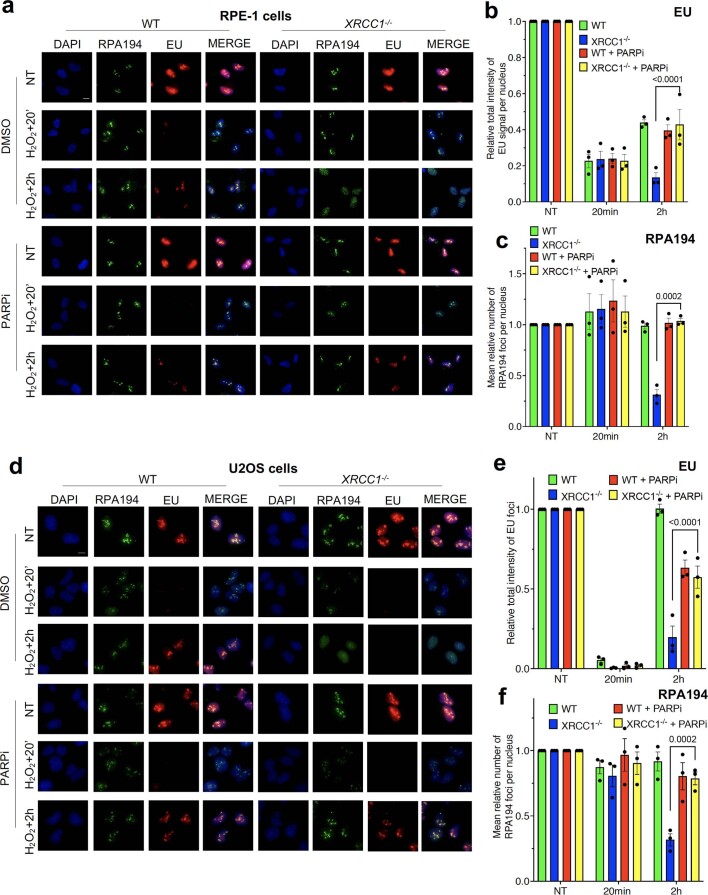
Reduced transcriptional recovery in XRCC1-deficient cells is a result of toxic PARP1 activity. **a**, Representative images of the RNAPI foci (RPA194 immunofluorescence) and levels of global transcription (EU immunofluorescence) in WT and *XRCC1*^*−/−*^ RPE-1 cells pretreated with PARP1 inhibitor (PARPi) following mock treatment or at the indicated times after treatment with 250 μM H_2_O_2_ for 5 min. Scale bars, 10 μm. **b** and **c**, Quantification of the RNAPI foci (RPA194) and levels of global transcription (EU immunofluorescence) shown in **(a)**. Data are means (±s.e.m.) of three independent experiments, and statistically significant differences were determined by two-way ANOVA with Tukey’s multiple comparisons test (p values are indicated). **d**, Representative images of the RNAPI foci (RPA194) and levels of global transcription (EU immunofluorescence) in WT and *XRCC1*^*−/−*^ U2OS cells pretreated with PARP1 inhibitor (PARPi) following mock treatment or at the indicated times after treatment with 1 mM H_2_O_2_ for 20 min. Scale bars, 10 μm. **e** and **f**, Quantification of the RNAPI foci (RPA194) and levels of global transcription (EU immunofluorescence) shown in **(d)**. Data are means (±s.e.m.) of three independent experiments, and statistically significant differences were determined by two-way ANOVA with Tukey’s multiple comparisons test (p values are indicated). Source data

**Extended Data Fig. 4:**
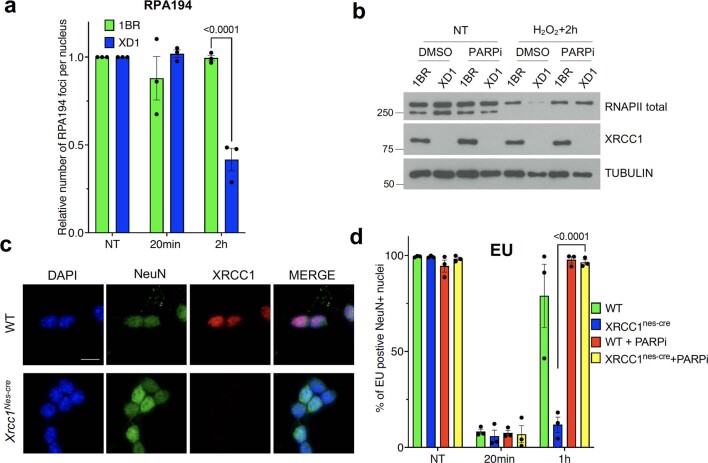
Toxic PARP1 activity suppresses transcriptional recovery in XRCC1 patient fibroblasts and Xrcc1-deficient mouse cerebellar neurons. **a**, Quantification of the RNAPI foci (RPA194) shown in **(**Fig. 3a**)**. Data are means (±s.e.m.) of three independent experiments, and statistically significant differences were determined by two-way ANOVA with Sidak’s multiple comparisons test (p values are indicated). **b**, Immunoblot of RNAPII hyperphosphorylation in normal fibroblasts (1BR) and XRCC1 patient fibroblasts (XD1) following mock treatment or 2 h after treatment with 60 μM H_2_O_2_ for 5 min. Cells were incubated with DMSO vehicle or with 10μM PARP inhibitor (PARPi) for 1 h prior to, during, and following H_2_O_2_ treatment as indicated. A representative image from one of three independent experiments is shown. **c**, XRCC1 and NeuN immunofluorescence in WT and *Xrcc1*^*nes-cre*^ mouse cerebellar neurons. Scale bars, 10 μm. **d**, Quantification of the levels of global transcription (EU immunofluorescence) shown in **(**Fig. 3c**)**. Data are means (±s.e.m.) of three independent experiments, and statistically significant differences were determined by two-way ANOVA with Tukey’s multiple comparisons test. Source data

Hereditary mutations in human XRCC1 result in cerebellar ataxia, a phenotype that is also observed in *Xrcc*^Nes-cre^ mice in which Xrcc1 is conditionally deleted in the brain^18,19,26^. This phenotype is largely rescued by the deletion of Parp1, the primary sensor of unrepaired SSBs in mammalian cells^18,19^. We therefore examined the impact of PARP1 deletion on the recovery of transcription in *XRCC1*^−/−^ cells. Strikingly, PARP1 deletion rescued the transcriptional recovery in *XRCC1*^−/−^ RPE-1 cells, as measured by EU pulse labelling, RNAPI foci and RNAPII phosphorylation (Fig. 2a–d). In addition, transcription recovery was rescued in both *XRCC1*^−/−^ RPE-1 and U2OS cells by incubation with PARP inhibitor (PARPi), confirming that the transcription defect was the result of toxic/aberrant ADP-ribosylation (Fig. 2e and Extended Data Fig. 3). Consistent with this idea, the defect observed in the *XRCC1*^−/−^ cells was recapitulated in WT RPE-1 cells by incubation with an inhibitor of PARG (the enzyme primarily responsible for poly(ADP-ribose) degradation), which suppressed the recovery of global transcription for up to 8 h following H_2_O_2_ treatment (Fig. 2f–h). Importantly, reduced transcriptional recovery following oxidative stress was also observed in patient-derived human fibroblasts with XRCC1 mutations as well as *Xrcc1*^Nes-cre^ mouse cerebellar neurones, and these defects were again prevented by PARPi, implicating PARP1-induced transcriptional repression in neurological disease (Fig. 3 and Extended Data Fig. 4).

### The poly(ADP-ribose)-binding activity of XRCC1 promotes transcriptional recovery by directly regulating PARP1 activity

**Fig. 4:**
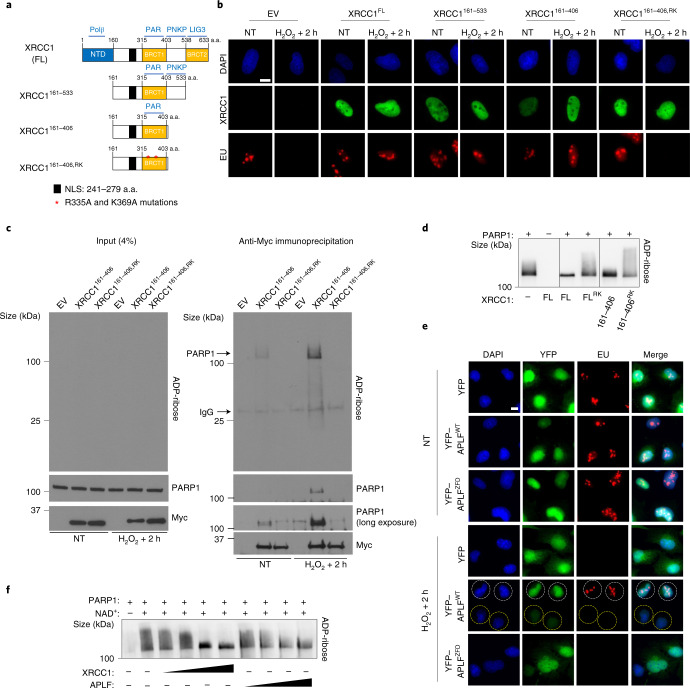
The poly(ADP-ribose)-binding activity of XRCC1 promotes transcriptional recovery following oxidative damage. **a**, Cartoon of full-length (FL) and truncated XRCC1 proteins encoded by the Myc- and/or His-tagged expression constructs employed in this work. The interaction partners are shown (top): Polβ, DNA polymerase β; PAR, poly(ADP-ribose); PNKP, polynucleotide kinase phosphatase; LIG3, DNA ligase III. The N-terminal domain (NTD), nuclear localization signal (NLS) and two BRCT domains are also shown; a.a., amino acids. **b**, Representative images showing the levels of global transcription (EU pulse labelling; bottom) in *XRCC1*^−/−^ U2OS cells, transiently transfected with expression constructs encoding the indicated XRCC1 proteins, following mock treatment or 2 h after treatment with 1 mM H_2_O_2_ for 20 min. The XRCC1 protein levels are also shown (middle). The cells were pulse labelled with EU as in Fig. 1. EV, empty vector. **c**, Immunoblot of Myc-tagged XRCC1 proteins, PARP1 and ADP-ribose levels in cell extract from the indicated transiently transfected *XRCC1*^−/−^ U2OS cells before (left, input) and after (right) anti-Myc immunoprecipitation, 2 h after mock treatment or treatment with 1 mM H_2_O_2_ for 20 min (right). **d**, XRCC1 suppresses PARP1 auto-ADP-ribosylation in vitro. Human recombinant PARP1 (100 nM) was incubated in the presence of single-stranded DNA (100 nM), NAD^+^(2.5 μM) and with or without 1 μM of full-length XRCC1 or the indicated XRCC1 protein fragment for 30 min. The reaction products were fractionated by SDS–PAGE and detected by western blotting with ADP-ribose detection reagent. **e**, Representative images showing the levels of global transcription (EU pulse labelling) in *XRCC1*^−/−^ U2OS cells stably transfected with expression constructs encoding WT APLF (yellow fluorescent protein (YFP)–APLF^WT^) or its ADP-ribose binding mutant (YFP–APLF^ZFD^) following mock treatment or 2 h after treatment with 1 mM H_2_O_2_ for 20 min. White and yellow circles indicate examples of cells over-expressing or not YFP-APLF, respectively. For **b**,**e**, representative images from one of three independent experiments are shown. Scale bars, 10 μm. **f**, APLF suppresses PARP1 auto-ADP-ribosylation in vitro. Human recombinant PARP1 (100 nM) was incubated in the presence of single-stranded DNA (100 nM), NAD^+^(2.5 μM) and with or without 0.25, 0.5, 1 or 1.5 μM WT recombinant human APLF or XRCC1 for 30 min. The reaction products were fractionated by SDS–PAGE and detected by western blotting with ADP-ribose detection reagent. **c**,**d**,**f**, Representative blots from one of three independent experiments are shown. Source data

**Extended Data Fig. 5:**
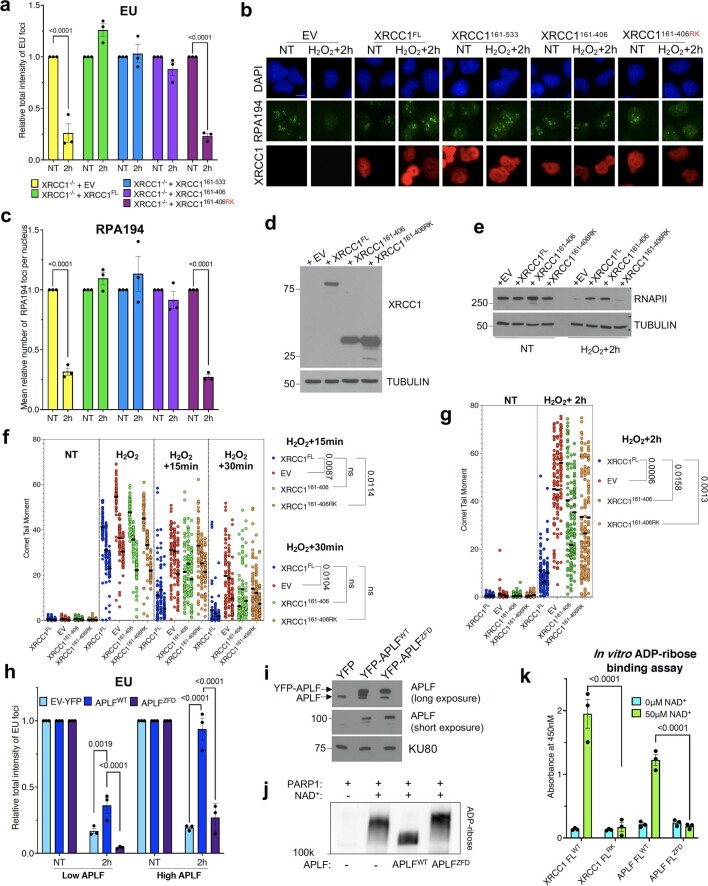
The poly(ADP-ribose) binding activity of XRCC1 promotes transcriptional recovery following oxidative damage. **a**, Quantification of the levels of global transcription (EU immunofluorescence) shown in Fig. 4b. Data are means (±s.e.m.) of three independent experiments, and statistically significant differences were determined by two-way ANOVA with Sidak’s multiple comparisons test (p values are indicated). **b**, Representative images of the RNAPI (RPA194) and XRCC1 immunofluorescence in WT U2OS cells, *XRCC1*^*−/−*^ U2OS cells, and *XRCC1*^*−/−*^ U2OS cells transiently transfected with empty vector (EV) or expression construct encoding the indicated XRCC1 proteins, following mock-treatment or 2 h after treatment with 1 mM H_2_O_2_ for 20 min. Scale bars, 10 μm. **c**, Quantification of the RNAPI foci (RPA194) shown in **(b)**. Data are means (±s.e.m.) of three independent experiments, and statistically significant differences were determined by two-way ANOVA with Sidak’s multiple comparisons test (p values are indicated). **d**, Immunoblot of XRCC1 in *XRCC1*^*−/−*^ U2OS cells stably expressing the indicated XRCC1 proteins. A representative blot from three independent experiments is shown. **e**, Immunoblot of RNAPII hyperphosphorylation in *XRCC1*^*−/−*^ U2OS cells stably expressing the indicated XRCC1 proteins, following mock-treatment or 2 h after treatment with 1 mM H_2_O_2_ for 20 min. A representative image from one of three independent experiments is shown. **f** and **g**, Quantification of DNA strand breaks measured by alkaline comet assays in *XRCC1*^*−/−*^ U2OS cells stably expressing the indicated XRCC1 proteins, following mock treatment (not treated; ‘NT’) or at the indicated times after treatment with either 50 μM H_2_O_2_ for 10 min on ice (conditions to measure SSB repair kinetic; panel f)or with 100 μM H_2_O_2_ for 20 min (refreshed every 10 min) in serum-free medium at 37 °C followed by recovery for 2 h (conditions, at which transcription recovery is perturbed in *XRCC1*^*−/−*^ cells; panel g). Data show individual tail moments (an arbitrary measure of DNA breaks) from 50 cells per sample for three independent experiments. For each sample, the fifty tail moments are stacked vertically and the three experiments are positioned side-by-side (exp1, exp2, exp3) with the tick mark centred on exp2. Statistical significance was determined by one way ANOVA with Sidak’s two tailed Student’s t-test (p values are indicated). **h**, Quantification of the levels of global transcription (EU immunofluorescence) shown in Fig. 4d. Data are means (±s.e.m.) of three independent experiments, and statistically significant differences were determined by two-way ANOVA with Tukey’s multiple comparisons test (p values are indicated). **i**, Immunoblot of APLF in the *XRCC1*^*−/−*^ U2OS cells stably expressing the indicated APLF proteins. Single validation of SDS–PAGE mobility shift. **j**, Immunoblot of PARP1 auto-ADP-ribosylation in an *in vitro* ADP-ribosylation assay. Human recombinant PARP1 (100 nM) was incubated for 30 min in the presence of ssDNA (100 nM), NAD^+^(2.5 μM) and with or without 1.5 μM of wild type or Zn-finger double mutant APLF (APLF^WT^; APLF^ZFD^ respectively). A representative blot from one of three independent experiments is shown. **k**, Binding of full length XRCC1 and APLF, as well as their PAR binding mutants denoted as XRCC1^RK^ and APLF^ZFD^ respectively, to the indicated mock-ribosylated (-NAD^+^) or ribosylated (50 μM NAD^+^) histones. Data are means (±s.e.m.) of three independent experiments, and statistically significant differences were determined by two-way ANOVA with Tukey’s multiple comparisons test (p values are indicated). Source data

To understand how XRCC1 prevents toxic PARP1 activity, we identified the protein domain/s important for this process. A truncated Myc-histidine (Myc-His)-tagged fragment of XRCC1 encoding only the central one-third of the protein (Myc-His–XRCC1^161–406^) rescued transcription recovery in *XRCC1*^−/−^ U2OS cells (Fig. 4a,b and Extended Data Fig. 5a–e). This was surprising because this fragment lacks the protein-interaction domains that underpin the role of XRCC1 as a molecular scaffold^27^ and so cannot support normal rates of SSB repair (Extended Data Fig. 5f,g). The ability of XRCC1 to bind directly to poly(ADP-ribose) was required to prevent PARP1-mediated transcriptional suppression, as indicated by the failure of a mutant derivative of Myc-His–XRCC1^161–406^ harbouring mutations that prevent binding to this polymer (R335A/K369A; denoted Myc-His–XRCC1^161–406,RK^)^9^ to rescue transcriptional recovery, thereby suggesting that XRCC1 might regulate poly(ADP-ribosylation) directly (Fig. 4b and Extended Data Fig. 5a–e). The most probable target of this regulation is PARP1 itself, given that this protein was the most abundant, if not the only, ribosylated protein detected in Myc-His–XRCC1^161–406^ immunoprecipitates following H_2_O_2_ treatment and its recovery was prevented by the mutations that prevent poly(ADP-ribose) binding (Fig. 4c). Consistent with direct regulation of PARP1 activity by the poly(ADP-ribose)-binding domain of XRCC1, both recombinant full-length XRCC1 and XRCC1^161–406^ reduced PARP1 auto-ribosylation in vitro, whereas the mutant derivatives of these proteins that cannot bind poly(ADP-ribose) were unable to do so (Fig. 4d).

These data suggest that XRCC1 can suppress aberrant PARP1 activity not only by accelerating SSB repair but also by direct binding to, and suppression of, poly(ADP-ribose) chain extension. To confirm this idea, we examined whether transcriptional recovery could be rescued by the overexpression of APLF, a structurally distinct poly(ADP-ribose)-binding protein that can also negatively regulate poly(ADP-ribose) synthesis^28^. Recombinant APLF suppressed poly(ADP-ribose) chain extension in vitro, albeit less effectively than XRCC1, and overexpression of high levels of APLF restored transcriptional recovery in *XRCC1*^−/−^ U2OS cells in a manner dependent on the poly(ADP-ribose)-binding zinc finger domain^29^ (Fig. 4e,f and Extended Data Fig. 5h–k). These data indicate that the negative regulation of PARP1 activity by direct binding of XRCC1 to poly(ADP-ribose) is both required and sufficient for normal transcription recovery following oxidative stress.

### Prolonged transcriptional suppression is triggered by low but persistent PARP1 activity at unrepaired DNA BER intermediates

**Fig. 5:**
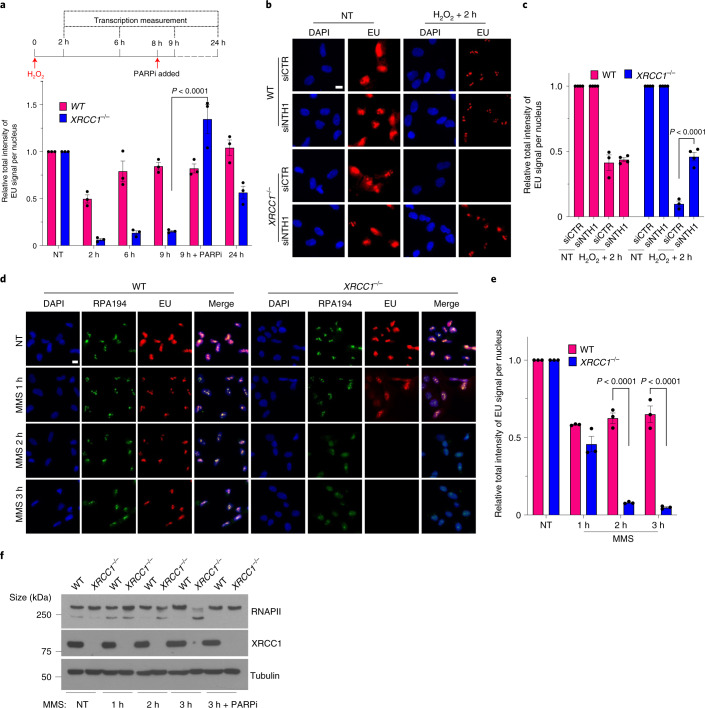
Unrepaired BER intermediates are a source of toxic PARP1 activity and transcriptional suppression in XRCC1-defective cells. **a**, Levels of global transcription (EU immunofluorescence; bottom) from the experiment outlined in the schematic (top; see also Extended Data Fig. 6a). **b**, Representative images of the levels of global transcription (EU pulse labelling) in WT and *XRCC1*^−/−^ RPE-1 cells following mock treatment or at 2 h after treatment with 250 μM H_2_O_2_ for 5 min. The cells were pretreated with control (siCTR) or siRNA targeting *NTH1* (siNTH1) for 72 h. The cells were pulse labelled with EU as in Fig. 1. **c**, Levels of global transcription from the experiment in **b**. **d**, Representative images of the levels of global transcription (EU pulse labelling) in WT and *XRCC1*^−/−^ RPE-1 cells following mock treatment or at the indicated times after treatment with 0.1 mg ml^−1^ MMS (continuous treatment). The cells were pulse labelled with EU as in Fig. 1. **b**,**d**, Scale bars, 10 μm. **e**, Levels of global transcription (EU pulse labelling) from the experiment shown in **d**. **a**,**c**,**e**, Data are the mean ± s.e.m. of three independent experiments. Statistical significance was determined using a two-way ANOVA with Sidak’s multiple comparisons test (significantly different *P* values are indicated). **f**, Immunoblot of RNAPII hyperphosphorylation in WT and *XRCC1*^−/−^ RPE-1 cells following mock treatment or after treatment for the indicated times with 0.1 mg ml^−1^ MMS. Where indicated, 10 μM PARPi was present throughout the MMS treatment. A representative blot from one of three independent experiments is shown. Source data

**Extended Data Fig. 6:**
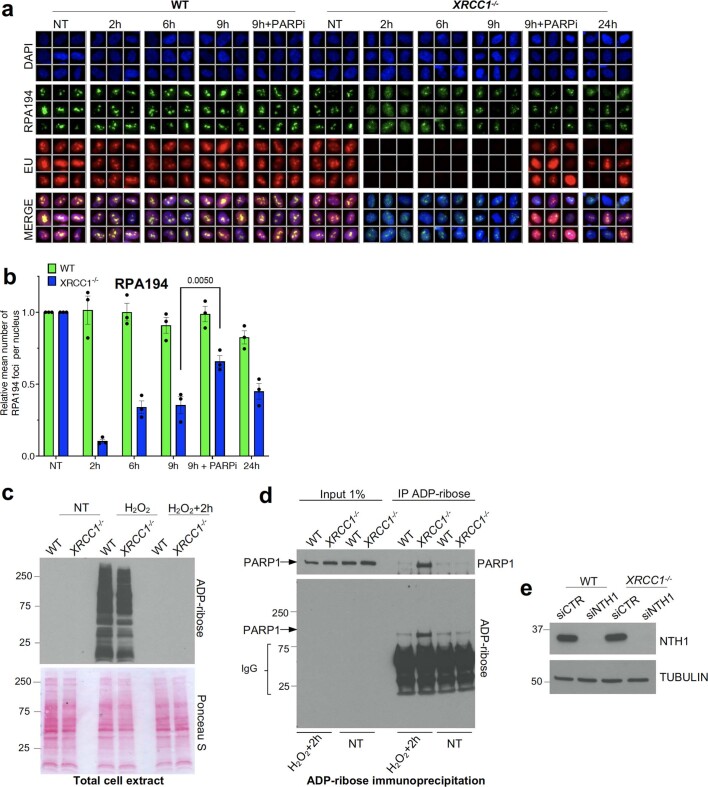
Persistent PARP1 activation on the BER intermediates is a source of toxic PARP1 activity. **a**, Representative images of the RNAPI foci (RPA194) and levels of global transcription (EU Immunofluorescence) in WT and *XRCC1*^*−/−*^ RPE-1 cells following mock treatment or at the indicated times after treatment with 250 μM H_2_O_2_ for 5 min. **b**, Quantification of the RNAPI foci (RPA194) shown in **(a)**. Data are means (±s.e.m.) of three independent experiments, and statistically significant differences were determined by two-way ANOVA with Sidak’s multiple comparisons test (p values are indicated). **c** and **d**, Immunoblot of PARP1 and/or ADP-ribose levels in total cell extract (panel **c**) and following ADP-ribose immunoprecipitation (panel **d**) prepared from WT and *XRCC1*^*−/−*^ RPE-1 cells following mock treatment or at 2 h after treatment with 250 μM H_2_O_2_ for 5 min. Representative blots from one of three independent experiments are shown. **e**, Immunoblot of NTH1 in WT and *XRCC1*^*−/−*^ RPE-1 cells treated for 72 h with 10 nM siRNA against *NTH1*. A representative blot from one of two independent experiments is shown. Source data

Collectively, the experiments described so far indicate that although the initial suppression of transcription by H_2_O_2_ is independent of PARP1 activity and probably reflects the stalling/inefficient bypass of DNA lesions by RNAPs^20–22^, the prolonged suppression observed in *XRCC1*^−/−^ cells is imposed by excessive/aberrant PARP1 activity at persistent SSBs. To address this further we conducted time-course experiments to determine when and for how long transcriptional suppression occurs. Whereas WT RPE-1 cells continued to recover transcription from 2 h following H_2_O_2_ treatment, with the transcription levels restored to normal within 24 h, *XRCC1*^−/−^ cells exhibited little or no transcriptional recovery for at least 9 h following H_2_O_2_ treatment (Fig. 5a and Extended Data Fig. 6a,b). Moreover, incubation with PARPi for 1 h rescued transcription even if applied 8 h after the H_2_O_2_ treatment, indicating that the failure to recover transcription in the *XRCC1*^−/−^ cells was a result of prolonged and ongoing ADP-ribosylation (Fig. 5a and Extended Data Fig. 6a,b). Consistent with this idea, although H_2_O_2_-induced protein ADP-ribosylation declined below detectable levels in both WT and *XRCC1*^−/−^ cell extracts 2 h after H_2_O_2_ treatment, as measured by western blotting, we detected low but persistent levels of PARP1 auto-ribosylation in the *XRCC1*^−/−^ cell extracts following enrichment with anti-ADP-ribose antibodies (Extended Data Fig. 6c,d).

We reasoned that the low level of persistent PARP1 activity in the *XRCC1*^−/−^ cells most probably reflected SSBs arising as intermediates of BER, given that while SSBs induced directly by disintegration of oxidized deoxyribose arise immediately, SSBs arising during BER require the enzymatic excision of oxidized DNA bases and thus arise more slowly. Moreover, we recently showed that PARP1 can become ‘trapped’ on SSB intermediates in *XRCC1*^−/−^ cells during BER and that low but persistent levels of PARP1 auto-ribosylation is a feature of this phenomenon^30^. Consistent with this idea, short interfering RNA (siRNA)-mediated depletion of NTH1—a DNA glycosylase that excises a broad spectrum of oxidized DNA bases^31–33^—rescued the recovery of transcription in H_2_O_2_-treated *XRCC1*^−/−^ cells (Fig. 5b,c and Extended Data Fig. 6e). Furthermore, global transcription was substantially suppressed in *XRCC1*^−/−^ cells following prolonged treatment with methyl methanesulfonate (MMS), an alkylating agent that induces SSBs largely or entirely via BER (Fig. 5d–f). This repression was also accompanied by a loss of RNAPII hyperphosphorylation, which once again was rescued by PARPi (Fig. 5f). Together, these data indicate that transcriptional recovery is suppressed in *XRCC1*^−/−^ cells by low levels of persistent PARP1 activity at unrepaired BER intermediates.

### Aberrant PARP1 activity disrupts global histone monoubiquitination

**Extended Data Fig. 7:**
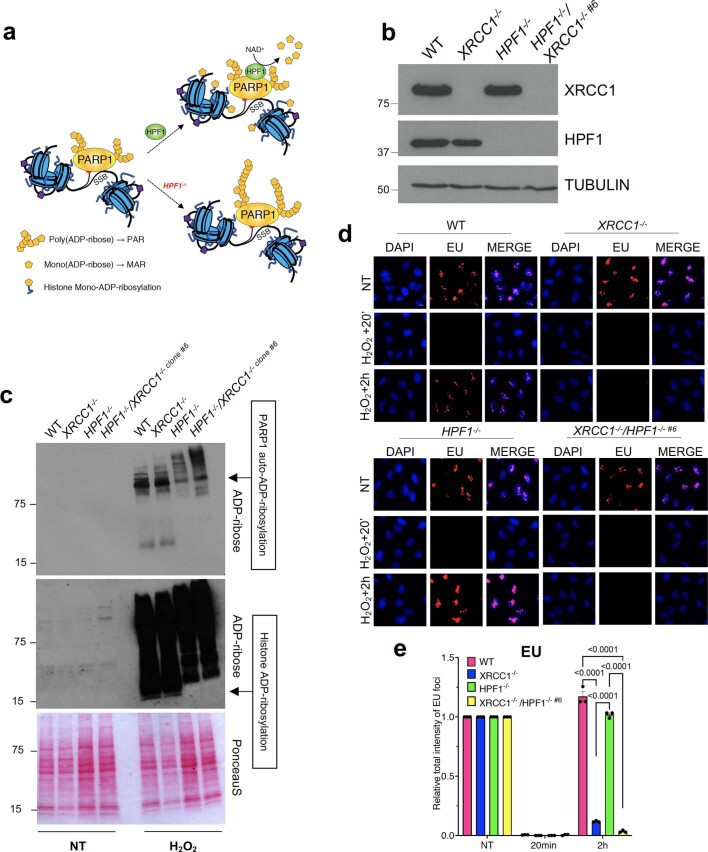
Deletion of the histone ADP-ribosylation factor HPF1 does not rescue transcription recovery in *XRCC1*^*−/−*^ RPE-1 cells. **a**, Model of the impact of HPF1 on PARP1-mediated histone ADP-ribosylation as well as PARP1 auto-ADP-ribosylation. **b**, Immunoblot of HPF1 and XRCC1 in WT, *HPF1*^*−/−*^, *XRCC1*^*−/−*^, and *HPF1*^*−/−*^*/XRCC1*^*−/−*^ U2OS cells. A representative blot from one of three independent experiments is shown. **c**, Immunoblot of ADP-ribosylation upon H_2_O_2_ treatment (1 mM, 5 min) in WT, *HPF1*^*−/−*^, *XRCC1*^*−/−*^, and *HPF1*^*−/−*^*/XRCC1*^*−/−*^ U2OS cells. A representative blot from one of three independent experiments is shown. **d**, Global transcription (EU Immunofluorescence) in WT, *HPF1*^*−/−*^, *XRCC1*^*−/−*^, *HPF1*^*−/−*^*/XRCC1*^*−/−*^ U2OS cells following mock treatment or at the indicated times after treatment with 1 mM H_2_O_2_ for 20 min. Scale bars, 10 μm. **e**, Quantification of the EU signal shown in **(d)**. Data are means (±s.e.m.) of three independent experiments, and statistically significant differences were determined by two-way ANOVA with Tukey’s multiple comparisons test (p values are indicated). Source data

Next, we examined the mechanism/s by which aberrant PARP1 activity inhibits transcription recovery during BER. Given that PARP1 can affect transcription by ADP-ribosylating histones^34–36^, we examined the impact of the deletion of histone parylation factor-1 (HPF1), a protein partner of PARP1 that is required for histone ADP-ribosylation^37–40^. However, although HPF1 deletion prevented detectable histone ADP-ribosylation in *XRCC1*^−/−^ U2OS cells as expected, it did not rescue transcription recovery, suggesting that histone ribosylation is not the cause of transcriptional suppression (Extended Data Fig. 7).

**Fig. 6:**
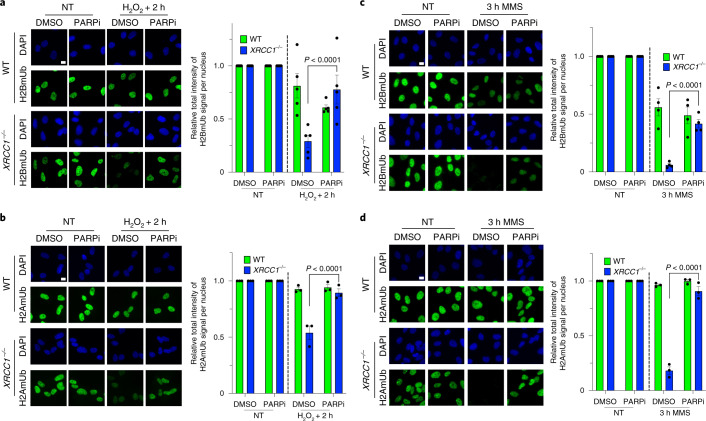
Toxic PARP1 activity during BER disrupts global histone monoubiquitination. **a**, Representative images (left) and levels (right) of H2BmUB in WT and *XRCC1*^−/−^ RPE-1 cells following mock treatment or at the indicated times after treatment with 250 μM H_2_O_2_ for 5 min. **b**, Representative images (left) and levels (right) of H2AmUb in WT and *XRCC1*^−/−^ RPE-1 cells following mock treatment or at the indicated times after treatment with 250 μM H_2_O_2_ for 5 min. **a**,**b**, The cells were incubated with DMSO vehicle or 10 μM PARPi for 1 h before, during and following H_2_O_2_ treatment as indicated. **c**, Representative images (left) and levels (right) of H2BmUB in WT and *XRCC1*^−/−^ RPE-1 cells following mock treatment or at the indicated times after treatment with 0.1 mg ml^−1^ MMS (continuous treatment). **d**, Representative images (left) and levels (right) of H2AmUb in WT and *XRCC1*^−/−^ RPE-1 cells following mock treatment or at the indicated times after treatment with 0.1 mg ml^−1^ MMS (continuous treatment). **c**,**d**, The cells were incubated with DMSO vehicle or 10 μM PARPi during the MMS treatment. **a**–**d**, Data are the mean ± s.e.m. of five (**a**), three (**b**,**d**) and four (**c**) independent experiments. Statistical significance was determined using a two-way ANOVA with Tukey’s multiple comparisons test (significantly different *P* values are indicated). Scale bars, 10 μm. Source data

**Extended Data Fig. 8:**
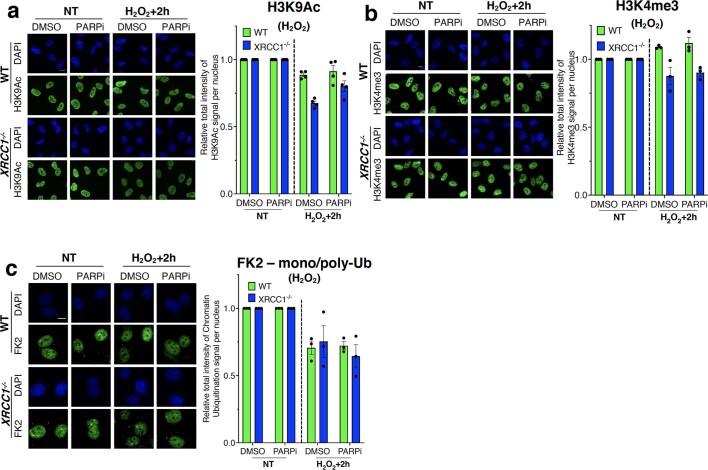
Levels of histone acetylation, methylation, protein ubiquitylation in *XRCC1*^−/−^ RPE-1 cells. **a**, Levels of H3K9Ac in RPE-1 cells, following treatment with 250 μM H_2_O_2_ for 5 min. Cells were incubated with 10 μM PARP inhibitor (PARPi) for 1 h prior to, during, and following H_2_O_2_ treatment. Quantification of H3K9Ac immunofluorescence shown on the right. Data are means (±s.e.m.) of four independent experiments. Scale bars, 10 μm. **b**, Levels of H3K4me3 in RPE-1 cells, following treatment with 250 μM H_2_O_2_ for 5 min. Cells were incubated with 10 μM PARP inhibitor (PARPi) for 1 h prior to, during, and following H_2_O_2_ treatment. Quantification of H3K4me3 immunofluorescence shown on the right. Data are means (±s.e.m.) of three independent experiments. Scale bars, 10 μm. **c**, Levels of chromatin mono/poly-ubiquitination (FK2) in RPE-1 cells, following treatment with 250 μM H_2_O_2_ for 5 min. Cells were incubated with 10 μM PARP inhibitor (PARPi) for 1 h prior to, during, and following H_2_O_2_ treatment. Quantification of chromatin mono/poly-ubiquitination immunofluorescence shown on the right. Data are means (±s.e.m.) of three independent experiments. Scale bars, 10 μm. Source data

As PARP1 can also affect transcription indirectly, via the recruitment of chromatin remodelling proteins^41,42^, we examined whether aberrant ADP-ribosylation affected the levels of histone marks known to promote transcriptional activity. Although the global levels of methylation and acetylation on histone H3 (H3K4me3 and H3K9Ac, respectively) were slightly, albeit not statistically significantly, reduced in *XRCC1*^−/−^ cells compared with WT cells 2 h after H_2_O_2_ treatment (Extended Data Fig. 8a,b), the global levels of H2AmUb and H2BmUb were dramatically reduced (Fig. 6a,b, respectively). The reduction in levels of H2BmUb was particularly noteworthy because this modification promotes transcriptional activity by a number of different mechanisms, including the direct relaxation of chromatin structure, facilitation of RNAP elongation and modulation of the activity of different transcriptional regulators^43–45^. Similar results were observed following MMS treatment, thereby confirming that the loss of histone monoubiquitination in *XRCC1*^−/−^ cells was a result of DNA base damage (Fig. 6c,d). Moreover, normal levels of histone ubiquitination were restored in *XRCC1*^−/−^ cells by incubation with PARPi, demonstrating that the loss of these important histones modifications was the result of aberrant ADP-ribosylation (Fig. 6a–d). This impact of aberrant PARP activity on histone ubiquitination was particularly pronounced given that we did not detect a reduction in total chromatin ubiquitination, as detected by the anti-ubiquitin antibody FK2 (Extended Data Fig. 8c).

### PARP1 suppresses histone monoubiquination and transcription recovery by triggering aberrant recruitment of the ubiquitin protease USP3

**Fig. 7:**
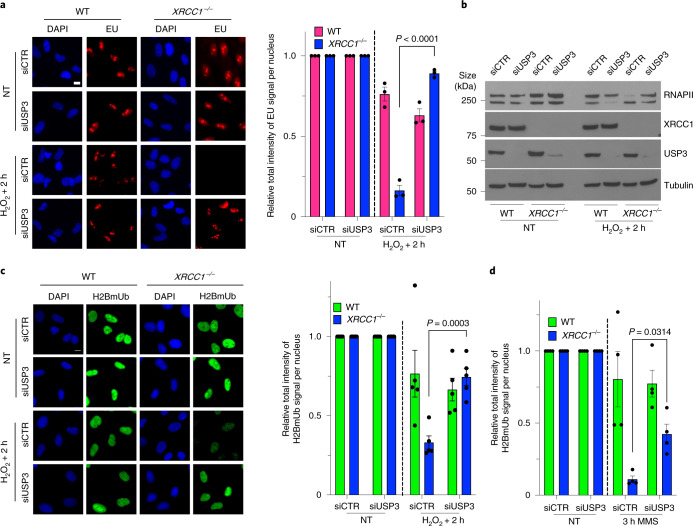
Toxic PARP1 activity disrupts histone monoubiquitination and promotes transcriptional suppression via USP3. **a**, Representative images (left) and levels (right) of global transcription (EU pulse labelling) in WT and *XRCC1*^−/−^ RPE-1 cells following mock treatment or at the indicated times after H_2_O_2_ treatment (250 μM; 5 min). The cells were pretreated with control (siCTR) or *USP3* siRNA (siUSP3) and pulse labelled with EU. **b**, RNAPII hyperphosphorylation in WT and *XRCC1*^−/−^ RPE-1 cells, pretreated with control or USP3 siRNA as indicated, following mock treatment or 2 h after treatment with H_2_O_2_ as in **a**. A representative blot of the whole-cell extracts from one of three independent experiments is shown. **c**, Representative images (left) and levels (right) of H2BmUb in WT and *XRCC1*^−/−^ RPE-1 cells, pretreated with control or *USP3* siRNA as in **a** following H_2_O_2_ treatment. **a**,**c**, Scale bars, 10 μm. **d**, Levels of H2BmUb in WT and *XRCC1*^−/−^ RPE-1 cells following mock treatment or treatment with 0.1 mg ml^−1^ MMS for 3 h. The cells were pretreated with control or *USP3* siRNA. **a**,**c**,**d**, Data are the mean ± s.e.m. of three (**a**), five (**c**) and four (**d**) independent experiments. Statistical significance was determined using a two-way ANOVA with Tukey’s multiple comparisons test (significantly different *P* values are indicated). Source data

**Fig. 8:**
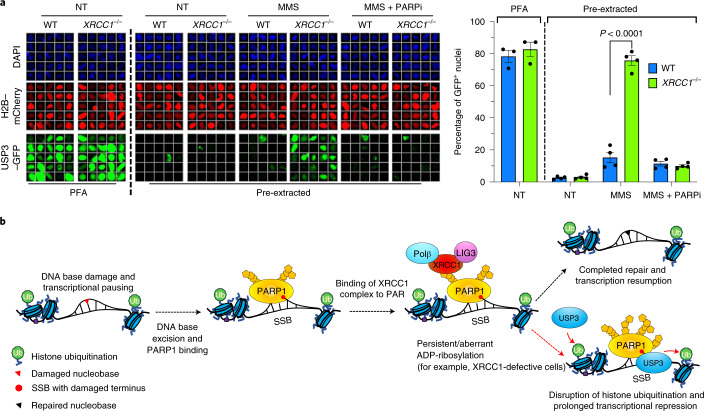
PARP1-dependent recruitment of USP3 into chromatin during BER in XRCC1-defective cells. **a**, Chromatin retention of USP3–GFP in WT and *XRCC1*^−/−^ U2OS cells transiently expressing H2B–mCherry and USP3–GFP following mock treatment or treatment with 0.1 mg ml^−1^ MMS in the presence or absence of 10 μM PARPi as indicated. The cells were either immediately fixed with paraformaldehyde (PFA) to detect total protein or first extracted with detergent to remove the soluble proteins (pre-extracted), as indicated. Data are the mean ±s.e.m. of four independent experiments. Statistical significance was determined using a two-way ANOVA with Sidak’s multiple comparisons test (significantly different *P* values are indicated). Representative scanR galleries of individual cells (left) and quantification (right) are shown. Scale bar, 10 μm. **b**, Model for prolonged PARP1/USP3-dependent suppression of transcription during BER. In WT cells, RNAP pausing at BER intermediates is accompanied by PARP1 activation, completion of BER by XRCC1 protein complexes and transcription resumption. In XRCC1-defective cells, persistent PARP1 activity at unrepaired BER intermediates leads to aberrant USP3 recruitment, extensive/excessive protein deubiquitination (including histones) at damaged and proximal/nearby undamaged sites, and prolonged transcriptional suppression. Note that PARP1 inhibition prevents USP3 recruitment and rescues global transcription recovery, most probably reflecting the eventual bypass of unrepaired SSBs by RNAP and/or transcription resumption at proximal/nearby undamaged sites.

**Extended Data Fig. 9:**
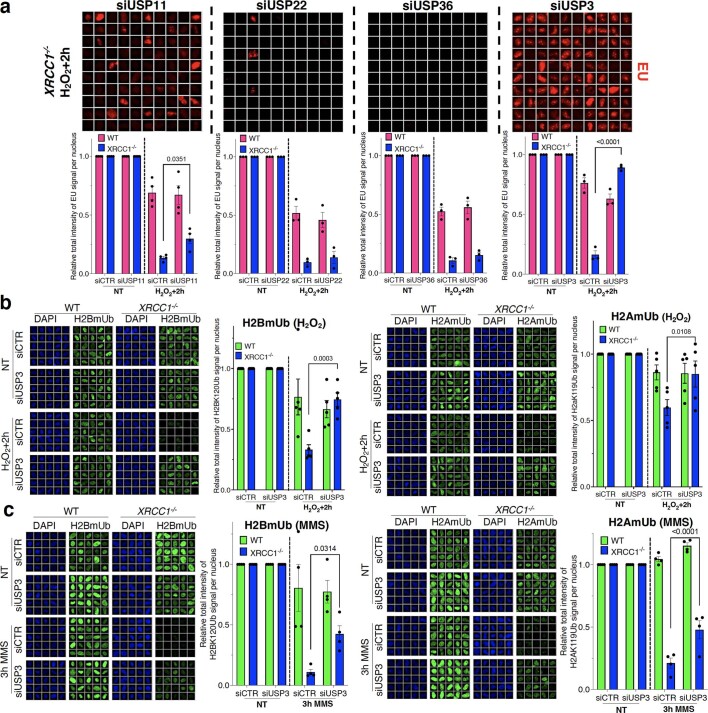
Activated PARP1 recruits USP3 to induce histone deubiquitination and transcription inhibition. **a**, Global transcription (EU Immunofluorescence) in RPE-1 cells, following treatment with 250 μM H_2_O_2_ for 5 min. Representative images of cells 2 h after H_2_O_2_ are shown. Cells were pre-treated for 72 h with control or siRNA targeting *USP11, USP22, USP36* and *USP3*. Quantifications are shown below images. Data are means (±SEM) of three or four independent experiments with statistical significance determined by Two-way ANOVA, Sidak’s multiple comparisons test (p values are indicated). **b**, Levels of H2BK120mUb (H2BmUb; left) and H2AK119mUb (H2AmUb; right) in RPE-1 cells, following treatment with 250 μM H_2_O_2_ for 5 min. Cells were pre-treated for 72 h with control or siRNA targeting *USP3*. Data are means (±SEM) of five independent experiments with statistical significance determined by two-way ANOVA with Sidak’s multiple comparisons test (p values are indicated). **c**, Levels of H2BmUb (left) and H2AmUb (right) in RPE-1 cells, following treatment with 0.1 mg/ml MMS for 3 h. Cells were pre-treated for 72 h with control or siRNA targeting *USP3*. Data are means (±SEM) of four independent experiments with statistical significance determined by two-way ANOVA with Sidak’s multiple comparisons test (p values are indicated). Source data

**Extended Data Fig. 10:**
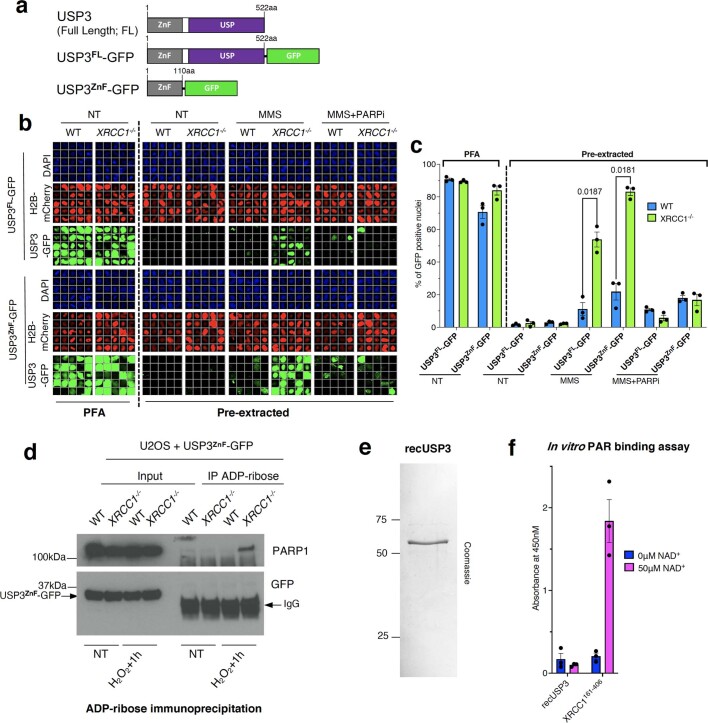
USP3 does not interact directly with Poly-ADP-ribose chains. **a**, Cartoon of GFP-tagged USP3 constructs employed in this work. **b**, Representative ScanR images of chromatin retention of USP3^**FL**^-GFP and USP3^**ZnF**^-GFP in WT and *XRCC1*^*−/−*^ U2OS cells, following mock-treatment or for 4 h with 0.1 mg/ml MMS in the presence/absence of 10 μM PARPi as indicated. Cells were transfected with H2B–mCherry and USP3–GFP constructs 24 h before experiment. Cells were fixed with PFA to show total protein levels or were pre-extracted with detergent prior to fixation to show chromatin-bound proteins. **c**, Quantification of experiments depicted in **b**. Data are means (±s.e.m.) of three independent experiments, and statistically significant differences were determined by two-way ANOVA with Sidak’s multiple comparisons test (p values are indicated). **d**, Immunoblot of PARP1 and USP3^**ZnF**^-GFP levels in anti-ADP-ribose immunoprecipitates from WT and *XRCC1*^*−/−*^ U2OS cell extracts following mock treatment or 1 h after treatment with 1 mM H_2_O_2_ for 20 min. A representative blot from one of two independent experiments is shown. **e**, Coomassie staining of purified recombinant human USP3 employed in the ADP-ribose binding assay shown in **f**. Single purification. **f**, Binding of full length USP3 and XRCC1^161–406^ (positive control) to calf thymus histones mock-ribosylated (-NAD^+^) or ADP-ribosylated (50 μM NAD^+^) with recombinant PARP1. Data are means (±s.e.m.) of three independent experiments. Source data

Finally, to identify the mechanism by which aberrant PARP1 activity reduces histone monoubiquitination, we depleted enzymes that are known to deubiquitinate histone H2A and/or H2B^46–53^. Whereas siRNA directed against either of the deubiquitylases *USP22* and *USP36* failed to rescue transcription recovery in *XRCC1*^−/−^ cells following H_2_O_2_ treatment, siRNA to *USP11* resulted in a small but significant rescue of transcription recovery (Extended Data Fig. 9a). More importantly, however, siRNA directed against *USP3* resulted in almost complete rescue of transcription recovery in *XRCC1*^−/−^ RPE-1 cells following H_2_O_2_ treatment, whether measured by EU pulse labelling (Fig. 7a and Extended Data Fig. 9a) or RNAPII hyperphosphorylation (Fig. 7b). Consistent with these data, *USP3* siRNA prevented the PARP1-dependent reduction in histone monoubiquitination following H_2_O_2_ treatment (Fig. 7c and Extended Data Fig. 9b). The *USP3* siRNA also reduced the loss of histone monoubiquitination in *XRCC1*^−/−^ cells during continuous treatment with MMS (Fig. 7d and Extended Data Fig. 9c) throughout the 3 h time course. We also detected the accumulation of green fluorescent protein (GFP)-tagged USP3 in the chromatin of the *XRCC1*^−/−^ cells during MMS treatment, which was prevented by treatment with PARPi, providing support for PARP1 activity as a source of aberrant USP3 recruitment during BER (Fig. 8a). Interestingly, the amino (N)-terminal zinc finger domain of USP3 was sufficient for PARP-dependent recruitment at BER intermediates. However, we were unable to detect any evidence of direct binding of this domain to poly(ADP-ribose) (Extended Data Fig. 10a–f), which suggests that PARP1 mediates USP3 recruitment indirectly, perhaps via its well-established role in regulating chromatin compaction^6,54,55^.

## Discussion

XRCC1 is a molecular scaffold protein that interacts with many of the enzymatic components of SSB repair^27^. At SSBs arising during BER, as obligate intermediates of base excision, XRCC1 interacts with and stabilizes DNA polymerase β and DNA ligase III^56^. Another critical role of XRCC1 during SSB repair is to prevent excessive activation of PARP1, which can otherwise lead to neurological dysfunction^18,19^. However, the mechanisms by which excessive/aberrant PARP1 activity triggers this pathology have been unclear. One recent clue emerged from the discovery that the assembly of DNA repair protein complexes by XRCC1 is necessary to prevent excessive engagement and activity of PARP1 during BER, which otherwise leads to PARP1 ‘trapping’ on BER intermediates and consequently their reduced access and repair by other BER enzymes^30^. The data presented in this study demonstrate that the low but persistent levels of PARP1 activity associated with this ‘trapped’ PARP1 in XRCC1-defective cells lead to prolonged transcriptional suppression. This delay in transcription recovery is not simply a result of slower BER because PARP inhibition by a chemical inhibitor or overexpression of either of two distinct poly(ADP-ribose)-binding domains is sufficient to rescue transcription recovery in XRCC1-defective cells, even in the continued presence of unrepaired BER intermediates. The recovery of transcription at BER intermediates can thus be separated mechanistically from the completion of BER. This result may indicate that although SSBs may initially impede RNAP progression, these lesions can eventually be bypassed, as long as PARP1 signalling at the unrepaired SSBs is suppressed. Alternatively, the prolonged suppression of transcription by PARP1 detected in our experiments may reflect an impact on RNAP initiation and/or elongation in *trans* at sites distal to unrepaired SSBs.

PARP1 is a dynamic regulator of gene activity and is able to either repress or activate gene transcription in response to different physiological stimuli^57–59^. PARP activity can regulate transcription in a number of ways, such as by relaxing higher-order chromatin compaction and/or regulating RNAP activity^60–62^. PARP1 can also recruit polycomb and NuRD transcriptional repressor complexes, thereby facilitating the initial suppression of transcription following DNA damage^41,42^. However, we did not detect a requirement for PARP1 for the initial suppression of transcription following DNA damage—the initial reduction in EU pulse labelling observed following H_2_O_2_ treatment was not prevented by PARP1 deletion or inhibition. Thus, in our experiments the initial suppression of transcription probably reflects a PARP-independent mechanism, such as the pausing of RNAPII by BER intermediates^21,63^.

A striking feature of our work is the discovery that excessive/aberrant PARP1 activity prolongs transcriptional suppression via recruitment of the ubiquitin protease USP3. We do not yet know how poly(ADP-ribose) synthesis recruits USP3 into chromatin at unrepaired BER intermediates. Although the N-terminal zinc finger of USP3 is sufficient for this recruitment, we have so far not detected binding of this domain to poly(ADP-ribose). PARP1 might therefore mediate USP3 recruitment indirectly, such as via its ability to regulate chromatin compaction. For example, the ability of poly(ADP-ribose) to relax chromatin structure directly^64,65^ or indirectly via the recruitment of one or more chromatin remodelling enzymes^41,42,66–68^ may allow the USP3 zinc finger to detect its ubiquitylated substrate/s^51^. USP3 deubiquitinates multiple substrates, including the histones H2A, γH2AX and H2B, and is implicated in the cellular response to DNA damage both in vitro and in vivo^51–53,69^. In particular, reduced USP3 levels are associated with increased levels of DNA breakage and DNA replication stress as well as slower S phase progression^51,52^. USP3 reduces the level of γH2AX and H2A ubiquitination at K13 and K15, which are key sites of RNF8- and RNF168-mediated ubiquitination that regulate the repair of DNA double-strand breaks^53,69,70^. However, these modifications are unlikely to explain the impact of USP3 on transcription repression in our experiments, which result from unrepaired SSBs. Moreover, these modifications are not known to regulate transcriptional activity.

USP3 also deubiquitinates H2A and H2B monoubiquitination at K119 and K120, respectively^51,52^, both of which are critical for normal transcriptional control and are regulated in response to DNA damage^42,71–74^. Of these two modifications, it is the loss of H2BmUb that is a more likely explanation for the prolonged transcriptional suppression observed in our experiments. This is because this modification promotes transcriptional activity, whereas loss of H2AmUb ought to increase transcription. We do not yet know why loss of H2BmUb might dominate the transcriptional response in our experiments but it may relate to the different mechanisms and/or kinetics by which H2AmUb and H2BmUb regulate transcription. For example, loss of H2BmUb can reduce RNAP progression directly, and thus rapidly, by affecting nucleosome unwinding mediated by the histone chaperone FACT ahead of RNAP^44^. In contrast, loss of H2AmUb may increase gene transcription more slowly via the progressive loss of polycomb-repressive complexes from chromatin^75^. Consistent with this idea, we did not detect changes in the levels of H3K4me3, which is a downstream target of H2AmUb^76^, in our experiments (Extended Data Fig. 8).

H2BmUb is tightly coupled with RNAP elongation^44,77,78^ and promotes transcription in several ways, including facilitating histone H3K4 and H3K79 methylation^79,80^ and cooperating with FACT^44^. H2BmUb is also regulated in response to DNA damage. For example, H2BmUb is introduced at DNA double-strand breaks as a result of ATM-mediated phosphorylation and recruitment of the RNF20–RNF40 heterodimer, the ubiquitin E3 ligase that promotes this modification^81^. More intriguingly, with respect to the current work, H2BmUb is deubiquitinated at sites of DNA damage that have stalled RNAP^82^. In yeast, H2BmUb is deubiquitinated by Ubp8 and Ubp10, the former of which is the ubiquitin protease most closely related to USP3. It is thus tempting to speculate that a similar process occurs at sites of stalled RNAP in human cells and that the prolonged transcriptional suppression by USP3 in BER-defective cells is a pathological extension of this process (Fig. 8b).

Finally, hereditary XRCC1 mutations result in cerebellar ataxia and deletion of XRCC1 in the mouse brain results in cerebellar ataxia, seizures and juvenile mortality^18,19,26^. The impact of excessive/aberrant PARP1 activity on these phenotypes is illustrated by PARP1 inhibition and/or deletion, which alleviate the neuropathology in Xrcc1-defective neurons and mice^19^. Xrcc1-defective neurons also exhibit a defect in pre-synaptic Ca^2+^ signalling, which is rescued by PARP inhibition/deletion, which in light of our recent data might reflect an impact of aberrant PARP1 activity on the expression of genes critical for normal neuronal activity^19^. Consistent with this idea, we recently demonstrated that neuronal enhancers are hotspots of SSB repair^83^, most probably as a result of cytosine demethylation during the epigenetic (re)programming of neuronal gene expression; a process in which XRCC1-mediated BER is strongly implicated^84–86^. It is also possible that PARP1 activity might impact on transcription in other pathophysiological conditions that induce DNA base damage, such as chronic inflammation^87^.

## Methods

### Cell lines, treatments and culture conditions

All cell lines were cultured in a low-oxygen (3%) incubator (37 °C and 5% CO_2_). RPE-1 cells (American Type Culture Collection) were cultured in DMEM-F12 GlutaMAX medium containing 10% FBS supplemented with penicillin–streptomycin. U2OS cells (American Type Culture Collection) were cultured in DMEM containing 10% FBS supplemented with penicillin–streptomycin and l-glutamine. Primary fibroblasts from patients with XRCC1 mutations (XD1)^18^ as well as control counterparts 1BR.3 (denoted as 1BR) were cultured in MEM medium containing 15% FBS supplemented with penicillin–streptomycin and l-glutamine. Unless stated otherwise, H_2_O_2_ (8.8 M stock; Sigma) was freshly diluted in serum-free medium (DMEM-F12 GlutaMAX) for each experiment and used at a final concentration of 60 μM for 5 min (XD1 fibroblasts), 250 μM for 5 min (RPE-1 cells and mouse cerebellar neurons) or 1 mM for 20 min with refreshment after 10 min (U2OS cells) in serum-free medium at 37 °C. Note that relatively frequent replacement of the H_2_O_2_ stock (2–3 months) was required for robust H_2_O_2_-induced inhibition of transcription. Following treatment, the cells were washed twice in complete medium to inactivate the H_2_O_2_ and harvested at the indicated time points. MMS (Sigma) was diluted in complete medium and employed at a final concentration of 0.1 mg ml^−1^ (0.01%). Where indicated, PARPi (KU0058948, Axon Medchem) was used at 10 μM for 1 h before the H_2_O_2_ treatment, and PARG inhibitor (Tocris) at 10 μM during and following H_2_O_2_ treatment, until the cells were harvested. MG132 (Tocris) and bortezomib (Millipore) were used at concentrations of 10 μM and 1 μM, respectively, for 1 h before and 2 h following H_2_O_2_ treatment until the cells were harvested. For the siRNA treatments, the cells were transfected 72 h before analysis with 10 nM of siRNA to *NTH1*, *USP11*, *USP22*, *USP36* or *USP3* (Supplementary Table 4) using RNAiMAX lipofectamine (Invitrogen).

*XRCC1*^−/−^, *PARP1*^−/−^ and *PARP1*^−/−^*XRCC1*^−/−^ RPE-1 and U2OS cells have been described/employed previously^8,18,88^. Derivatives of these cell lines harbouring expression constructs (Supplementary Table 2) encoding full-length or truncated recombinant *XRCC1* (ref. ^89^) or *APLF*^90^ were generated by co-transfection with a plasmid encoding puromycin resistance. Stably transfected cells were selected with puromycin (2 μg ml^−1^) and single colonies were isolated, amplified and verified by western blotting after one week. For the transient transfection experiments, U2OS cells were incubated with poly(ethyleneimine) solution (Sigma) for 6 h in serum-free medium and the cells were fixed and analysed after 24 h. *HPF1*^−/−^ and *XRCC1*^−/−^*HPF1*^−/−^ U2OS cells were generated by transfection of WT U2OS or *XRCC1*^−/−^ U2OS cells with a Cas9–GFP plasmid (Addgene., 48138) and plasmids encoding *HPF1* guide RNA (gRNA) no. 1 and no. 2, targeting the first exon of the *HPF1* gene (a gift from I. Ahel^38^; Supplementary Table 4), at a 1:1:1 ratio using Lipofectamine LTX (Invitrogen). *USP3*^−/−^ and *XRCC1*^−/−^*USP3*^−/−^ RPE-1 cells were generated as described above, with the use *USP3* gRNA no. 1 and 2 (Supplementary Table 4). Transfected cells were sorted (BD FACSMelody) for GFP^+^ cells two days after transfection and seeded into 96-wells plates for the amplification of single-cell clones, which were subsequently validated by immunoblotting. Gene editing was confirmed by Sanger sequencing.

Primary cerebellar neurons were established from seven-day-old (P7) male and female WT and Xrcc1^Nes-Cre^ (ref. ^18^) mouse pups using the Papain Dissociation System (Worthington). The mice from which these neurons were obtained were maintained in accordance with the UK Animal (Scientific Procedures) Act 1986 and satisfied local institutional regulations at the University of Sussex and under the auspices of UK Home Office project licence number P3CDBCBA8. The cells were seeded onto poly-l-lysine-coated coverslips at 6 × 10^5^ cells per well and maintained in Neurobasal A medium (Gibco) supplemented with B27 (Invitrogen), 5.25 mM KCl, 0.5 mM glutamine and penicillin–streptomycin in a humidified incubator (3% O_2_ and 5% CO_2_ at 37 °C). The cultures were employed for transcription experiments between 4 and 11 d in vitro.

### Western blotting

RPE-1 cells were harvested and lysed in TEB150 lysis buffer (50 mM HEPES pH 7.4, 150 mM NaCl, 2 mM MgCl_2_, 5 mM EGTA pH 8, 1 mM dithiothreitol (DTT), 0.5% Triton X-100 and 10% glycerol) containing protease inhibitor cocktail (Roche), phosphatase inhibitor cocktail (PhosSTOP, Sigma) and 1:1,000 benzonase (Sigma) for 45 min on ice. Lysis was stopped by the addition of SDS–PAGE sample buffer and boiling for 5 min at 90 °C. The other cell lines were harvested directly in sample buffer and boiled as described for the RPE-1 cells. The protein extracts were fractionated by SDS–PAGE, transferred to nitrocellulose, blocked in 0.1% TBS–Tween containing 5% non-fat dried milk and incubated with the indicated appropriate antibodies (Supplementary Table 1).

### ADP-ribose immunoprecipitation

Cells were harvested by trypsinization and lysed in TEB250 lysis buffer (50 mM HEPES pH 7.4, 250 mM NaCl, 2 mM MgCl_2_, 5 mM EGTA pH 8, 1 mM DTT, 0.5% Triton X-100, 10% glycerol, protease inhibitor cocktail (Roche), phosphatase inhibitors cocktail (PhosSTOP, Sigma), 50 μM PARPi, 100 μM PARG inhibitor and benzonase (Sigma; 1:1,000)) for 45 min on ice, followed by centrifugation (15 min at 16,000*g*). For immunoprecipitation, 0.5 mg or more of the protein lysate was incubated with antibody to mono/poly-ADP-ribose antibody (Cell Signalling; Supplementary Table 1) for 2 h and then with Protein G Dynabeads (Invitrogen) for 2 h. The beads were then washed (3×) with lysis buffer, resuspended in sample buffer and heated for 5 min at 90 °C and subjected to SDS–PAGE. The proteins were transferred to nitrocellulose and immunoblotted with ADP-ribose-binding reagent (Millipore; Supplementary Table 1).

### Immunofluorescence

For immunofluorescence, cells were fixed in 4% PFA in PBS for 10 min, permeabilized in 0.2% Triton X-100 solution in PBS for 10 min, rinsed in PBS and blocked in 5% BSA solution. For the purpose of H2BmUb staining, the cells were fixed in 100% methanol at room temperature for 10 min, rinsed in PBS and blocked in 5% BSA solution. The cells were incubated with the appropriate primary antibody (Supplementary Table 1) for 1 h, washed in PBS, incubated with the appropriate fluorophore-conjugated secondary antibody (Invitrogen) for 1 h and then counterstained with DAPI. For global chromatin ubiquitination (staining with antibody to FK2), the cells were pre-extracted on ice for 2 min with 0.2% Triton X-100 in PBS, rinsed quickly in cold PBS and fixed in 4% PFA in PBS. To measure the global sites of nascent RNA synthesis, the cells were pulse labelled in medium containing 1 mM EU (Abcam) for 20 min before fixation. The EU pulse-labelled cells were subjected to click chemistry using a Click-iT kit (Invitrogen) according to the manufacturer’s protocol. Click chemistry was performed after the blocking step of the immunofluorescence protocol described above. All images for quantitation were acquired using the automated wide-field microscope scanR image acquisition and analysis software, with >500 cells scored per sample (Olympus). Representative images were acquired with the use a wide-field microscope (Apotome; Zeiss). Where displayed, scanR cell galleries were generated by the Olympus Analysis Software as a representative example of the cell populations that were quantified.

### DNA strand-break measurements

To measure DNA strand breaks under the conditions at which transcription is inhibited in *XRCC1*^−/−^ cells, the cells were treated with 100 μM H_2_O_2_ for 20 min (the H_2_O_2_ was refreshed after 10 min) in serum-free medium at 37 °C and then in drug-free medium for 2 h (Extended Data Fig. 4g). To measure the kinetics of SSB repair, the cells were treated with 50 μM H_2_O_2_ for 10 min on ice and then in drug-free medium at 37 °C for the indicated time periods (Extended Data Fig. 4f). The DNA strand breaks were measured using alkaline comet assays, as described previously^91^, using the Comet Assay IV software (Perceptive Instruments).

### Protein purification

N-terminal His-tagged APLF and APLF^ZFD^ (harbouring the mutations C379A and C385A, and C421A and C427A in the PAR-binding zinc fingers ZF1 and ZF2, respectively)^28^ were expressed from the pET16b-APLF^90^ and pET16b-APLF^ZFD^ plasmids in BL21(DE3) *Escherichia coli* (NEB) by overnight induction with 0.5 mM isopropylthiogalactoside (IPTG) at 20 °C. Full-length C-terminal His-tagged XRCC1 and XRCC1^RK^ were expressed from pET16b-XH (ref. ^89^) and pET16b-XH^RK^ (ref. ^18^), and N-terminal His-tagged XRCC1^161–406^ and XRCC1^161–406RK^ from pTWO-E-His–XRCC1^161–406^ and pTWO-E-His–XRCC1^161-406RK^ (ref. ^9^) by induction with 1 mM IPTG in Luria–Bertani medium at 30 °C for 3 h. N-terminal His-tagged human USP3 was expressed from pET16b-USP3 in BL21(DE3) *E. coli* (NEB) by overnight induction with 0.5 mM IPTG at 20 °C. The proteins were purified by metal-chelate affinity chromatography and gel filtration before the buffer was exchanged to 150 mM NaCl, 50 mM Tris–HCl pH 7.5, 10% glycerol and 1 mM DTT. Aliquots were then snap-frozen in liquid nitrogen and stored at −80 °C.

### In vitro analysis of PARP1 auto-ribosylation

Recombinant high-specific-activity PARP1 (100 nM; Trevigen) was either mock ribosylated in the absence of NAD^+^ or auto-ribosylated in the presence of 2.5 μM NAD^+^ (NEB) and the indicated recombinant proteins. The reactions were carried out in 50 mM Tris–HCl pH 8, 0.8 mM MgCl_2_, 1% glycerol, 1.5 mM DTT and 100 nM of single-stranded oligodeoxyribonucleotide (5′-CATATGCCGGAGATCCGCCTCC-3′; Eurogentec), in a final volume of 20 μl at room temperature for 30 min. The reaction products were heated to 90 °C for 3 min in sample buffer and subjected to SDS–PAGE. The proteins were transferred to nitrocellulose and immunoblotted with ADP-ribose binding reagent (Millipore; Supplementary Table 1).

### USP3 expression construct

To create the USP3–GFP and USP3^ZnF(1–110)^–GFP expression constructs, *USP3* complimentary DNA (Source Bioscience) was amplified with the use of the indicated primers (Supplementary Table 4) and cloned in-frame into the BamHI and XhoI restriction sites of the pEGFP-N1 vector (Addgene).

### USP3 chromatin retention assay

U2OS cells were transiently transfected with USP3–GFP or USP3^ZnF(1–110)^–GFP and H2B–mCherry constructs 24 h before the experiment. The cells were then treated with 0.01 mg ml^−1^ MMS for 4 h in the presence or absence of 10 μM PARPi. The cells were washed once with PBS before incubation in CSK buffer (10 mM PIPES, pH 7.0, 100 mM NaCl, 300 mM sucrose, 3 mM MgCl_2_ and 0.7% Triton X-100) for 3 min at room temperature. The cells were subsequently washed in PBS and fixed in 4% PFA for 10 min. Next, the cells were counterstained with DAPI, mounted in Moviol and subjected to immunofluorescence analysis. Between 50 and 100 H2B–mCherry-expressing cells were counted per condition.

### In vitro ADP-ribose binding assay

First, 96-well plates were washed with H_2_O and then incubated overnight with 0.1 mg ml^−1^ of calf thymus histones (Sigma) at 4 °C. Next, the plates were blocked with 5% BSA in PBST (PBS containing 0.1% Tween-20) for 30 min. The absorbed histones were mock ADP-ribosylated in the absence of NAD^+^ or ADP-ribosylated in the presence of 50 μM NAD^+^ (Sigma) in PARP1 reaction buffer (50 mM Tris–HCl pH 8, 0.8 mM MgCl_2_, 1% glycerol and 1.5 mM DTT) containing 40 nM single-stranded oligodeoxyribonucleotide (5′-CATATGCCGGAGATCCGCCTCC-3′) and 10 nM high-specific-activity PARP1 (Trevigen) for 2 h. After subsequent washes with PBST, 500 nM of XRCC1-His, XRCC1-His^RK^, His–XRCC1^161–406^, His–APLF, His–APLF^ZFD^ and His–USP3 were added and incubated for 30 min on ice in Dilution buffer (20 mM Tris–HCl pH 7.5 and 130 mM NaCl). The plates were washed with PBST and incubated for 30 min with Dilution buffer containing mouse anti-polyhistidine antibody (Sigma) followed by three washes with PBST and subsequent incubation with horseradish peroxidase-conjugated secondary antibody (DAKO) for 30 min. After washing out the secondary antibody, 3,3′,5,5′-tetramethylbenzidine liquid substrate (slow kinetic form; Sigma) was added to the wells for 5 min. The reactions were stopped by the addition of 0.2 M HCl. Absorbance was read at 450 nm using a CLARIOstar microplate reader (BMG Labtech).

### Statistics and reproducibility

No statistical method was used to predetermine the sample size, but we routinely employed at least three biological repeats for each experiment, in each case scoring as many technical replicates as possible (typically several hundred/thousand cells) using the applicable automated microscope software (scanR or Comet Assay IV). All replicates were successful and included in the data. No data were excluded from the analyses. The samples/experiments were not randomized because all samples were specific genetic cell lines or samples derived from such and so randomization was not appropriate. The investigators were not blinded to allocation during the experiments and outcome assessment because all numerical data were software automated and independent of investigator subjectivity. All data presented in the manuscript are represented as the mean ± s.e.m., unless stated otherwise, and were analysed using GraphPad Prism (version 8/9). The statistical test employed for each dataset has been specified in the figure legends.

### Reporting Summary

Further information on research design is available in the Nature Research Reporting Summary linked to this article.

## Online content

Any methods, additional references, Nature Research reporting summaries, source data, extended data, supplementary information, acknowledgements, peer review information; details of author contributions and competing interests; and statements of data and code availability are available at 10.1038/s41556-021-00792-w.

## Supplementary information

Reporting Summary

Supplementary Tables 1–4 Supplementary Table 1: Antibodies used in this study and their application concentrations. Supplementary Table 2: Plasmids used in this study. Supplementary Table 3: Cell lines generated in this study. Supplementary Table 4: siRNA, CRISPR guide and primer sequences used in this study.

Peer Review Information

## Data Availability

Because of their large number, all microscope quantification datasets from this work, such as Excel and scanR/comet fluorescence quantification tables, are available from the corresponding author on reasonable request, citing the experiments of interest. Source data are provided with this paper.

## Source data

Source Data Fig. 1 Statistical source data.
Source Data Fig. 1 Unprocessed western blots.
Source Data Fig. 2 Statistical source data.
Source Data Fig. 2 Unprocessed western blots.
Source Data Fig. 3 Statistical source data.
Source Data Fig. 4 Unprocessed western blots.
Source Data Fig. 5 Statistical source data.
Source Data Fig. 5 Unprocessed western blots.
Source Data Fig. 6 Statistical source data.
Source Data Fig. 7 Statistical source data.
Source Data Fig. 7 Unprocessed western blots.
Source Data Extended Data Fig. 1 Statistical source data.
Source Data Extended Data Fig. 1 Unprocessed western blots.
Source Data Extended Data Fig. 2 Statistical source data.
Source Data Extended Data Fig. 2 Unprocessed western blots.
Source Data Extended Data Fig. 3 Statistical source data.
Source Data Extended Data Fig. 4 Statistical source data.
Source Data Extended Data Fig. 4 Unprocessed western blots.
Source Data Extended Data Fig. 5 Statistical source data.
Source Data Extended Data Fig. 5 Unprocessed western blots.
Source Data Extended Data Fig. 6 Statistical source data.
Source Data Extended Data Fig. 6 Unprocessed western blots.
Source Data Extended Data Fig. 7 Statistical source data.
Source Data Extended Data Fig. 7 Unprocessed western blots.
Source Data Extended Data Fig. 8 Statistical source data.
Source Data Extended Data Fig. 9 Statistical source data.
Source Data Extended Data Fig. 10 Statistical source data.
Source Data Extended Data Fig. 10 Unprocessed western blots.
