# Supplementary material for: XRCC1 protects transcription from toxic PARP1 activity during DNA base excision repair
Source: Nat Cell Biol. 2021 Nov 22;23(12):1287–98. doi: 10.1038/s41556-021-00792-w (PMC8683375; doi:10.1038/s41556-021-00792-w)
Supplement: Supplementary file 1 — Reporting Summary [file 41556_2021_792_MOESM1_ESM.pdf]

nature portfolio

Corresponding author(s): keith caldecott

Last updated by author(s): May 21, 2021

Reporting Summary

Nature Portfolio wishes to improve the reproducibility of the work that we publish. This form provides structure for consistency and transparency in reporting. For further information on Nature Portfolio policies, see our Editorial Policies and the Editorial Policy Checklist.

Statistics

For all statistical analyses, confirm that the following items are present in the figure legend, table legend, main text, or Methods section.

n/a	Confirmed	
☐	☒	The exact sample size (*n*) for each experimental group/condition, given as a discrete number and unit of measurement
☐	☒	A statement on whether measurements were taken from distinct samples or whether the same sample was measured repeatedly
☐	☒	The statistical test(s) used AND whether they are one- or two-sided<br>*Only common tests should be described solely by name; describe more complex techniques in the Methods section.*
☒	☐	A description of all covariates tested
☒	☐	A description of any assumptions or corrections, such as tests of normality and adjustment for multiple comparisons
☐	☒	A full description of the statistical parameters including central tendency (e.g. means) or other basic estimates (e.g. regression coefficient) AND variation (e.g. standard deviation) or associated estimates of uncertainty (e.g. confidence intervals)
☐	☒	For null hypothesis testing, the test statistic (e.g. *F*, *t*, *r*) with confidence intervals, effect sizes, degrees of freedom and *P* value noted<br>*Give P values as exact values whenever suitable.*
☒	☐	For Bayesian analysis, information on the choice of priors and Markov chain Monte Carlo settings
☒	☐	For hierarchical and complex designs, identification of the appropriate level for tests and full reporting of outcomes
☒	☐	Estimates of effect sizes (e.g. Cohen's *d*, Pearson's *r*), indicating how they were calculated

Our web collection on statistics for biologists contains articles on many of the points above.

Software and code

Policy information about availability of computer code

Data collection	scanR software, Comet Assay IV software,Graphpad Prism 9
Data analysis	scanR software, Comet Assay IV software,Graphpad Prism 9

For manuscripts utilizing custom algorithms or software that are central to the research but not yet described in published literature, software must be made available to editors and reviewers. We strongly encourage code deposition in a community repository (e.g. GitHub). See the Nature Portfolio guidelines for submitting code & software for further information.

Data

Policy information about availability of data

All manuscripts must include a data availability statement. This statement should provide the following information, where applicable:

- Accession codes, unique identifiers, or web links for publicly available datasets
- A description of any restrictions on data availability
- For clinical datasets or third party data, please ensure that the statement adheres to our policy

Data availability: Source data are provided with this study. Because of their large number,all raw microscope quantification data sets involved in this work such as Excel and scanR/comet fluorescence quantification tables (which generated the provided source data) are available from the corresponding author on reasonable request, citing the experiments of interest.

Field-specific reporting

Please select the one below that is the best fit for your research. If you are not sure, read the appropriate sections before making your selection.

☒ Life sciences ☐ Behavioural & social sciences ☐ Ecological, evolutionary & environmental sciences

For a reference copy of the document with all sections, see nature.com/documents/nr-reporting-summary-flat.pdf

Life sciences study design

All studies must disclose on these points even when the disclosure is negative.

Sample size	No statistical method was used to predetermine sample size, but we routinely employed at least three biological repeats for each experiment, in each case scoring as many technical replicates as possible (typically several hundred/thousand cells) using the applicable automated microscope software (scanR/comet assay IV).
Data exclusions	no data was excluded
Replication	all findings were independently replicated at least 3 times. All replicates were successful and included in the data
Randomization	this is not appropriate because all of our samples/experiments were specific genetically defined cell lines
Blinding	Samples/investigators were not blinded during experiments and outcome assessment because all numerical data was software automated and independent of investigator subjectivity.

Reporting for specific materials, systems and methods

We require information from authors about some types of materials, experimental systems and methods used in many studies. Here, indicate whether each material, system or method listed is relevant to your study. If you are not sure if a list item applies to your research, read the appropriate section before selecting a response.

Materials & experimental systems

n/a	Involved in the study	
☐	☒	Antibodies
☐	☒	Eukaryotic cell lines
☒	☐	Palaeontology and archaeology
☐	☒	Animals and other organisms
☒	☐	Human research participants
☒	☐	Clinical data
☒	☐	Dual use research of concern

Methods

n/a	Involved in the study	
☒	☐	ChIP-seq
☒	☐	Flow cytometry
☒	☐	MRI-based neuroimaging

Antibodies

Antibodies used

see below
Primary abs:
Antibody: Provider: Use: Cat. No.: Species:
XRCC1 Novus IF 1:300 WB 1:8000 NBP1-87154 Rabbit
TUBULIN Sigma WB 1:20000 T6074 Mouse
PARP1 BioRad WB 1:1000 MCA1522G Mouse
RNAPII Santa Cruz WB 1:100 sc-9001 Rabbit
RNAPII Santa Cruz WB 1:100 sc-55492 Mouse
NeuN Millipore IF 1:500 MAB377 Mouse
RNAPII pS2 Abcam WB 1:30000 ab5095 Rabbit
RNAPII pS5 Abcam WB 1:30000 ab5131 Rabbit
ADP-ribose binding reagent Millipore IF 1:1000 WB 1:10000 MABE1016 Rabbit
RPA194 Santa Cruz IF 1:200 WB 1:100 sc-48385 Mouse
NTH1 Santa Cruz WB 1:200 sc-130644 Mouse
HPF1 Novus WB 1:1000 NBP1-93973 Rabbit
H2BK120mUb Cell Signaling IF 1:800 5546T Rabbit
H2AK119mUb Cell Signaling IF 1:1000 8240T Rabbit
H3K9Ac Cell Signaling IF 1:1000 9649T Rabbit
H3K4me3 Abcam IF 1:400 Ab8580 Rabbit
USP11 Proteintech WB 1:1000 10244-1-AP Rabbit
FK2 BioRad IF 1:300 MCA6035 Mouse

KU80 Invitogene WB 1:10000 MA5-12933 Mouse

Secondary abs:
Alexa Fluor 488 Invitogene IF 1:1000 A21206 Donkey,
(anti-Rabbit)
Alexa Fluor 488 Invitogene IF 1:1000 A11001 Goat,
(anti-Mouse)
Alexa Fluor 647 Invitogene IF 1:1000 A21245 Goat,
(anti-Rabbit)
Alexa Fluor 647 Invitogene IF 1:1000 A21235 Goat,
(anti-Mouse)
APLF Invitogene WB 1:1000 PAS39226 Rabbit
USP3 Abcam WB 1:500 Ab229348

Validation	antibodies were chosen based on published literature/recommendation and were either not further validated or were internally validated by the use of cell lines lacking the target antigen

Eukaryotic cell lines

Policy information about cell lines

Cell line source(s)	RPE1 cells and U2OS cells were both from ATCC. Mouse cerebellar neurons were generated from mice in-house and human fibroblasts from our previous work (Hoch et al Nature 2017)
Authentication	these are all authenticated by fingerprinting in our cell culture facility
Mycoplasma contamination	all cells were confirmed mycoplasma negative.
Commonly misidentified lines (See ICLAC register)	no commonly misidentified cell lines were employed in this study

Animals and other organisms

Policy information about studies involving animals; ARRIVE guidelines recommended for reporting animal research

Laboratory animals	no live laboratory animals were used in this study. We used only isolated neurones.
Wild animals	no wild animals were employed
Field-collected samples	not applicable
Ethics oversight	we are governed by the UK Home Office and by the University Ethics committee. Our animal and research licenses are fully approved by such.

Note that full information on the approval of the study protocol must also be provided in the manuscript.
