# Supplementary figures and images for: XRCC1 protects transcription from toxic PARP1 activity during DNA base excision repair
Source: Nat Cell Biol. 2021 Nov 22;23(12):1287–98. doi: 10.1038/s41556-021-00792-w (PMC8683375; doi:10.1038/s41556-021-00792-w)

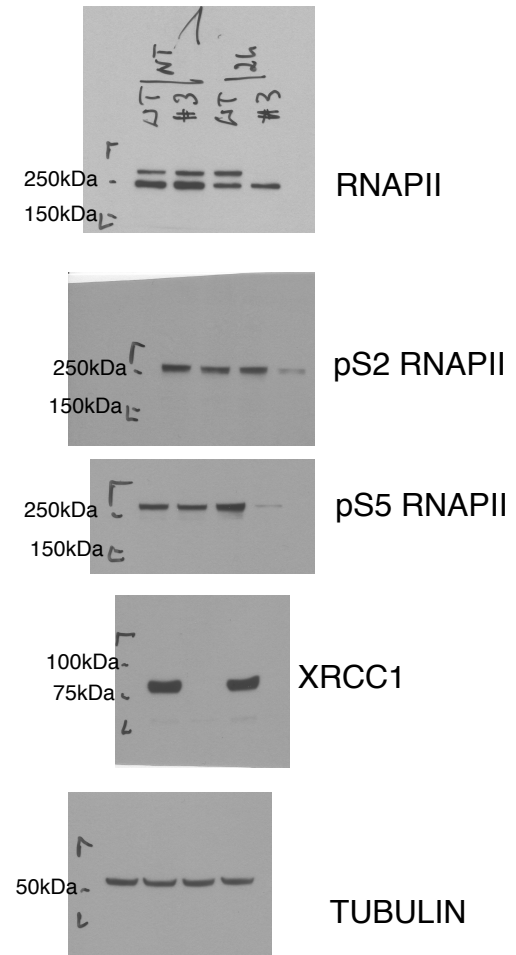

**Fig.1d**

Supplement: Source Data Fig. 1 — Unprocessed western blots. [file 41556_2021_792_MOESM5_ESM.pdf]

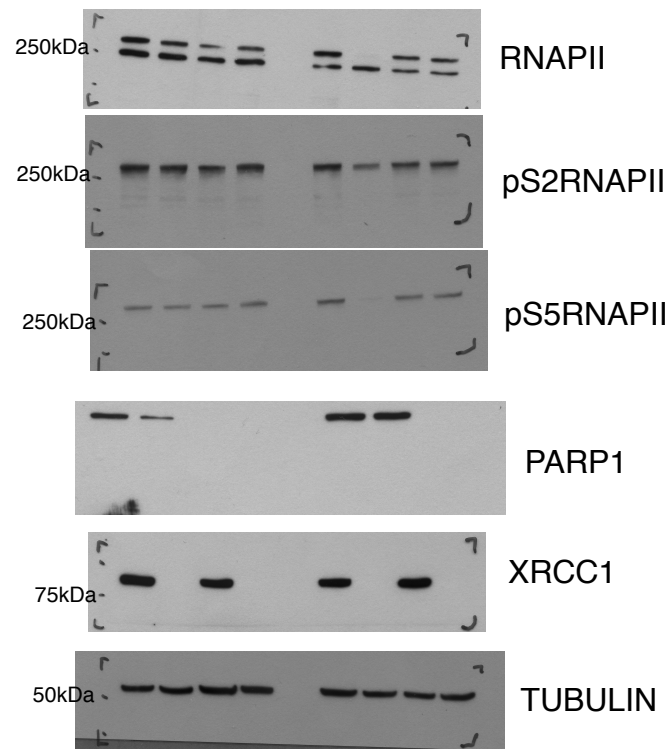

**Fig.2d**

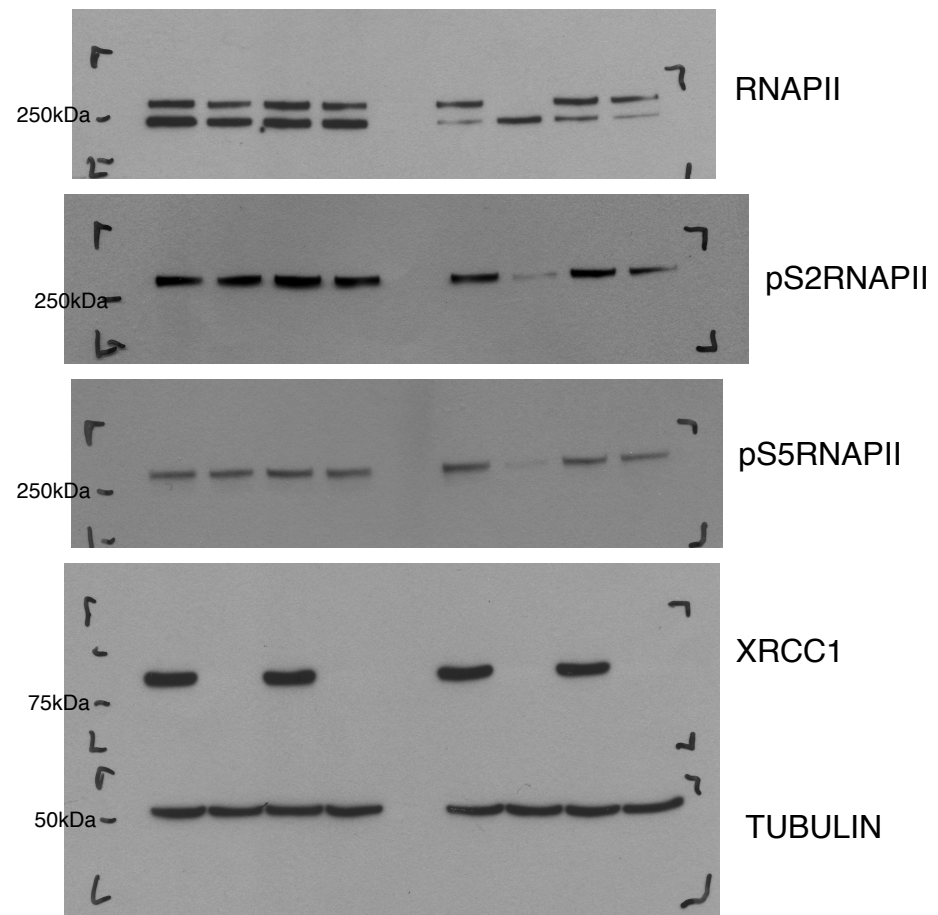

**Fig.2e**

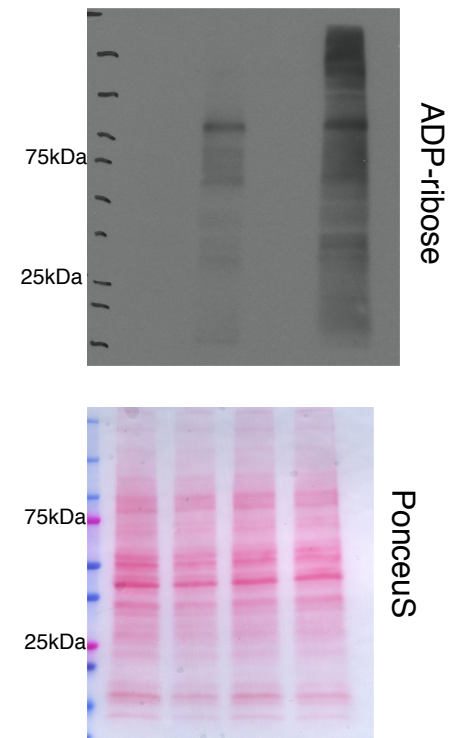

**Fig.2f**

Supplement: Source Data Fig. 2 — Unprocessed western blots. [file 41556_2021_792_MOESM7_ESM.pdf]

# INPUT

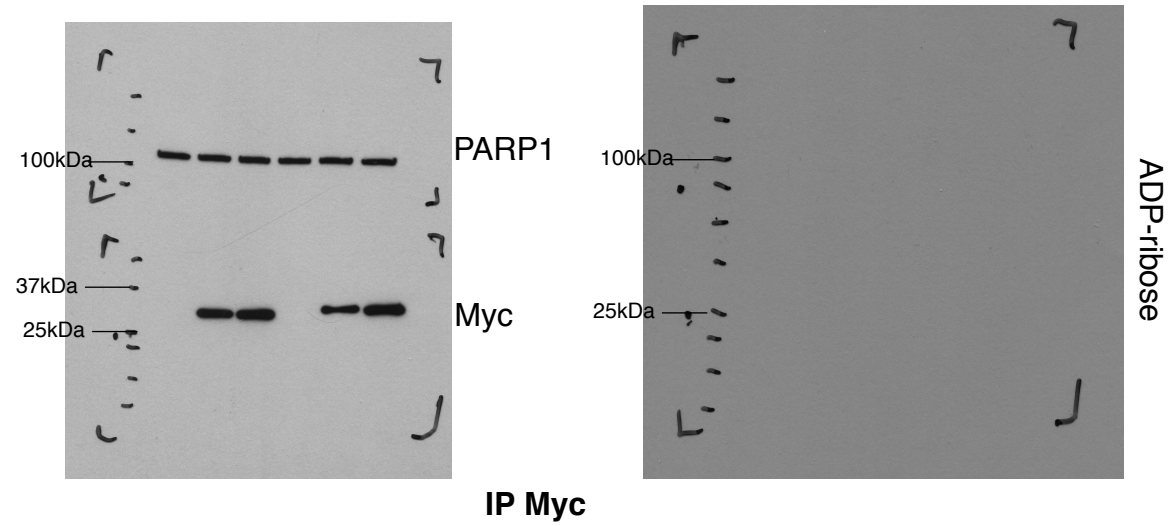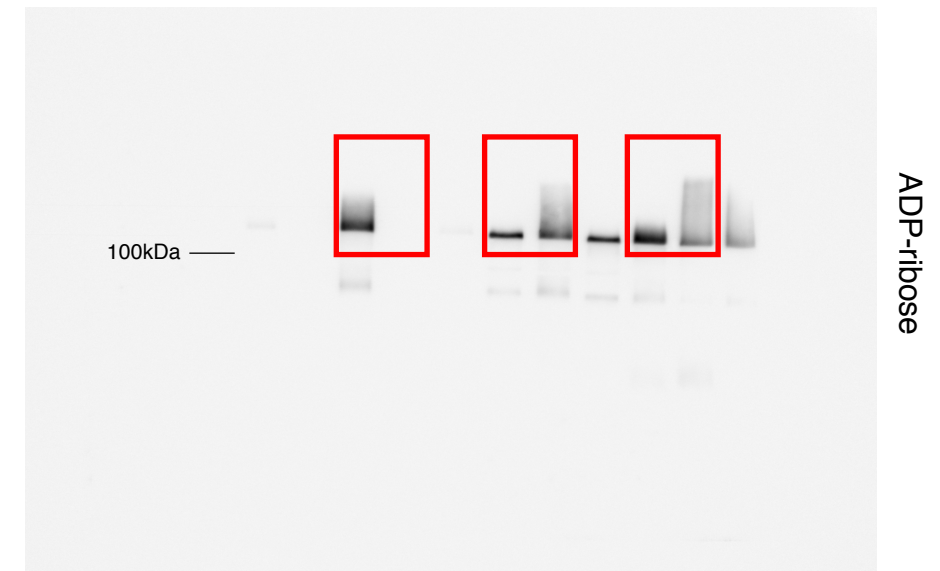

Fig.4d

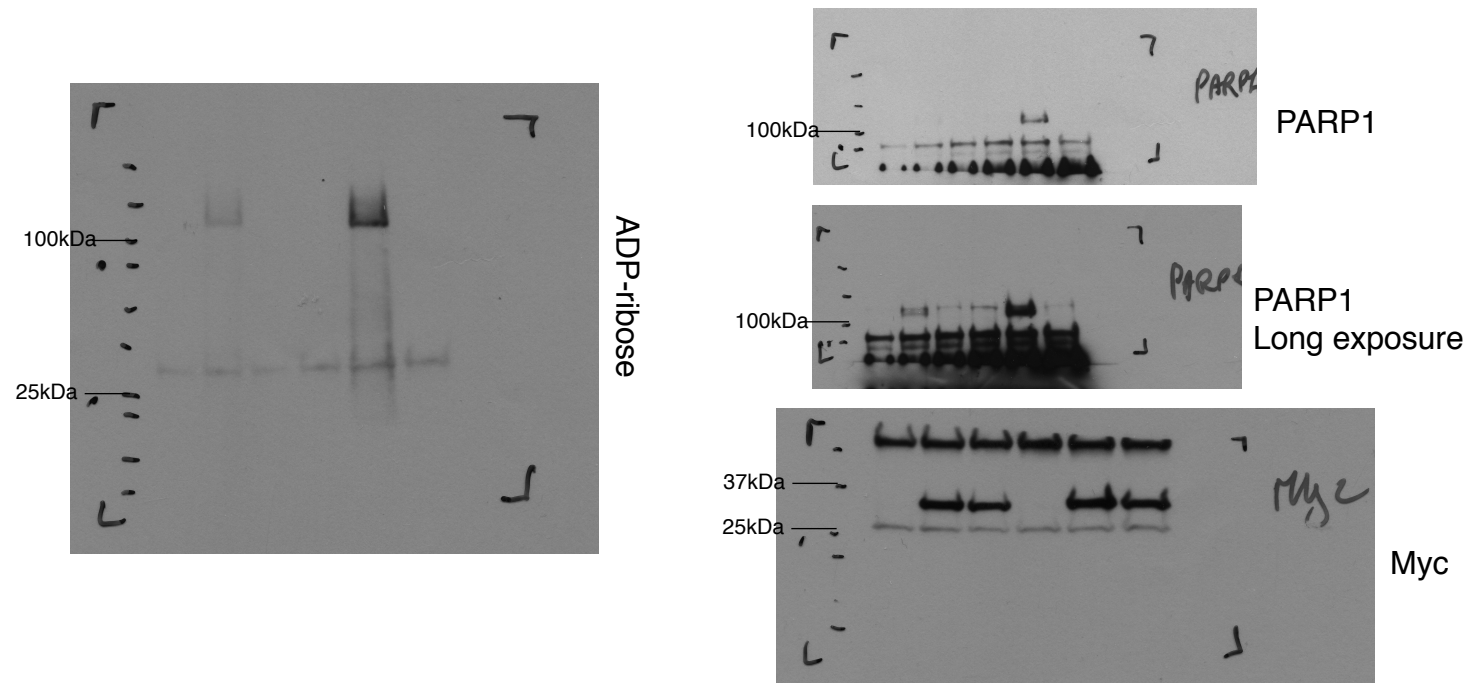

Fig.4c

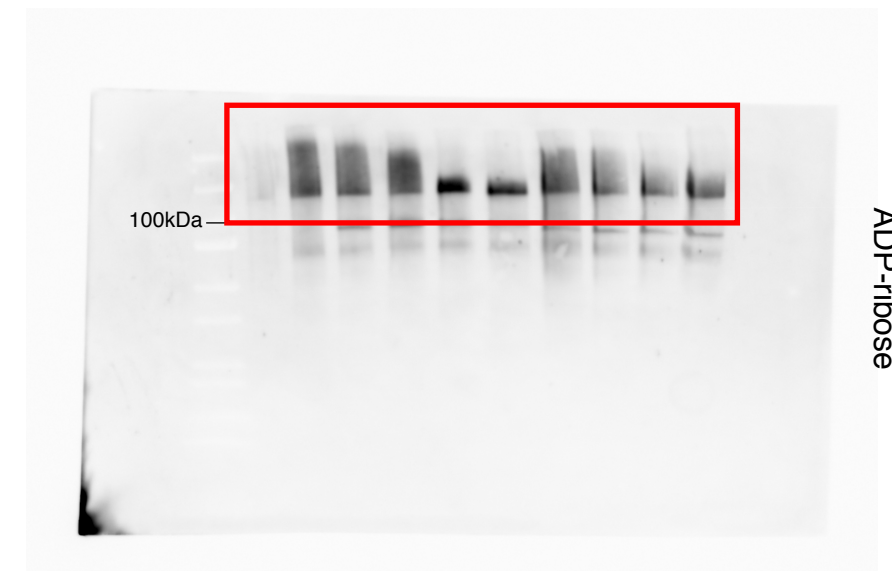

Fig.4f

Supplement: Source Data Fig. 4 — Unprocessed western blots. [file 41556_2021_792_MOESM9_ESM.pdf]

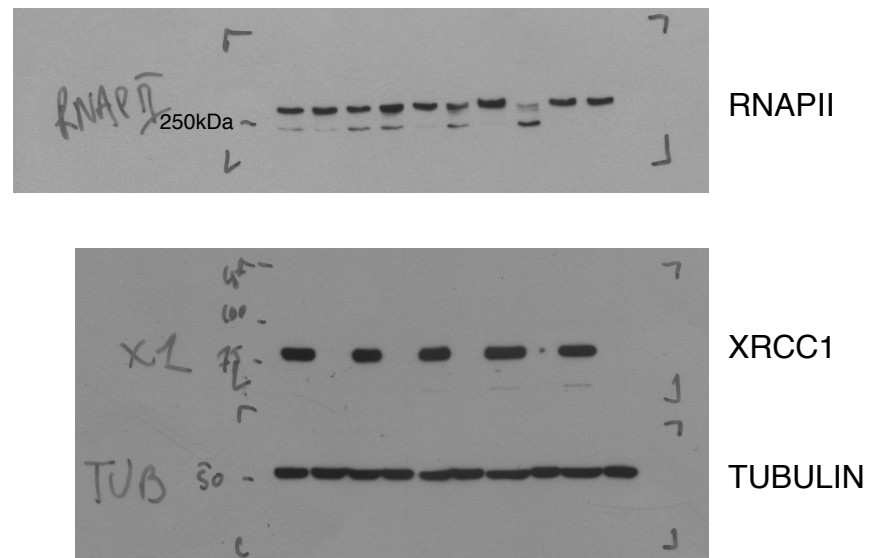

**Fig.5f**

Supplement: Source Data Fig. 5 — Unprocessed western blots. [file 41556_2021_792_MOESM11_ESM.pdf]

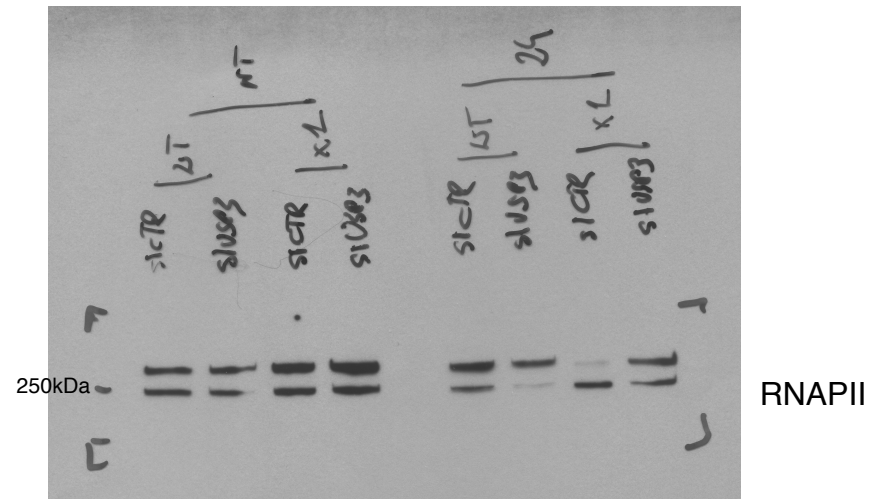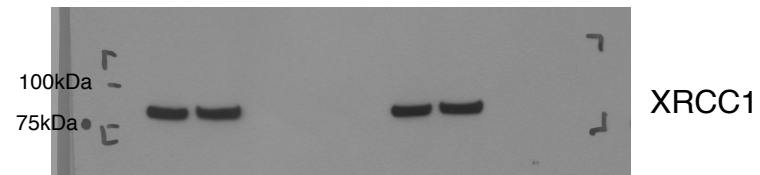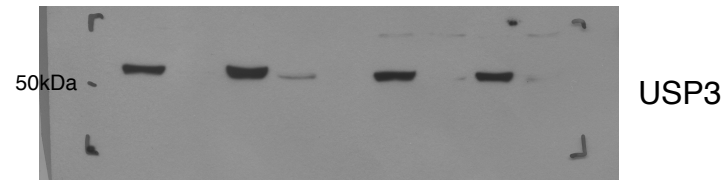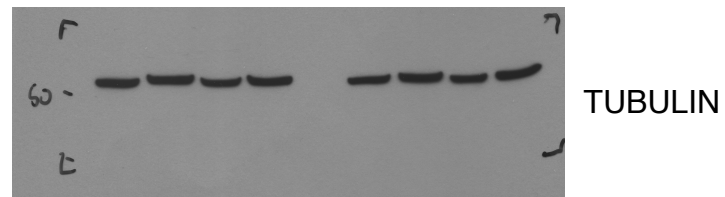

Fig.7b

Supplement: Source Data Fig. 7 — Unprocessed western blots. [file 41556_2021_792_MOESM14_ESM.pdf]

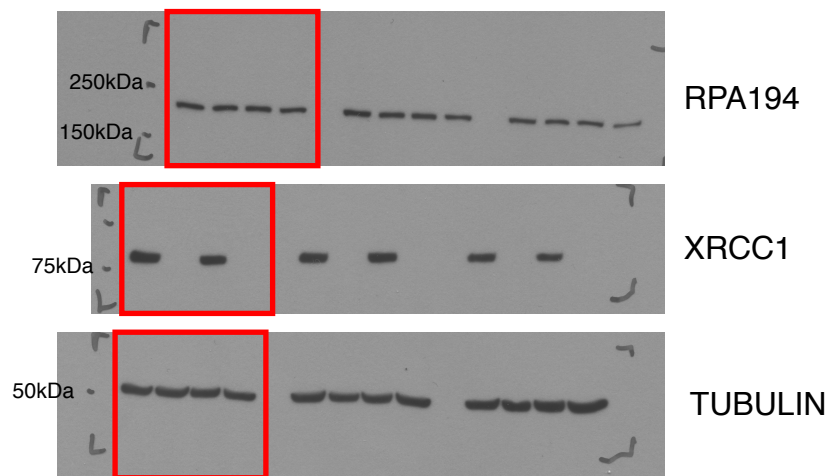

Extended Data Fig.1a

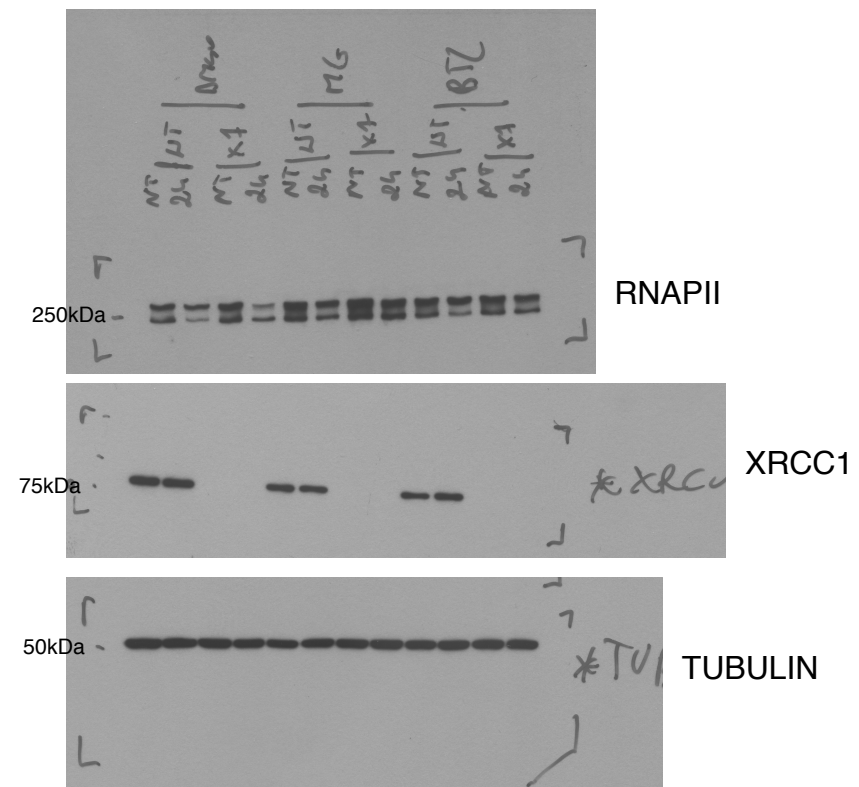

Extended Data Fig.1b

Supplement: Source Data Extended Data Fig. 1 — Unprocessed western blots. [file 41556_2021_792_MOESM16_ESM.pdf]

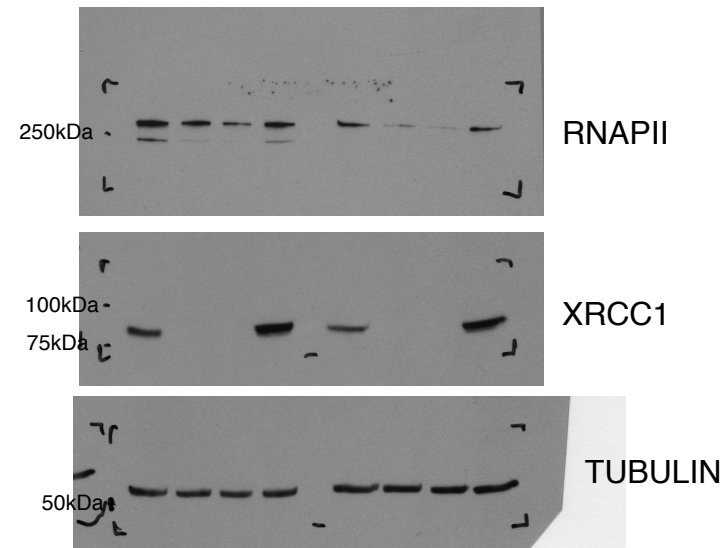

Extended Data Fig.2d

Supplement: Source Data Extended Data Fig. 2 — Unprocessed western blots. [file 41556_2021_792_MOESM18_ESM.pdf]

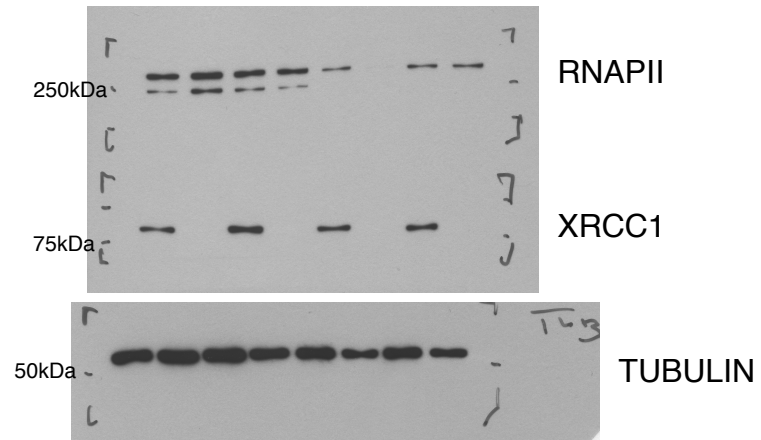

**Extended Data Fig.4b**

Supplement: Source Data Extended Data Fig. 4 — Unprocessed western blots. [file 41556_2021_792_MOESM21_ESM.pdf]

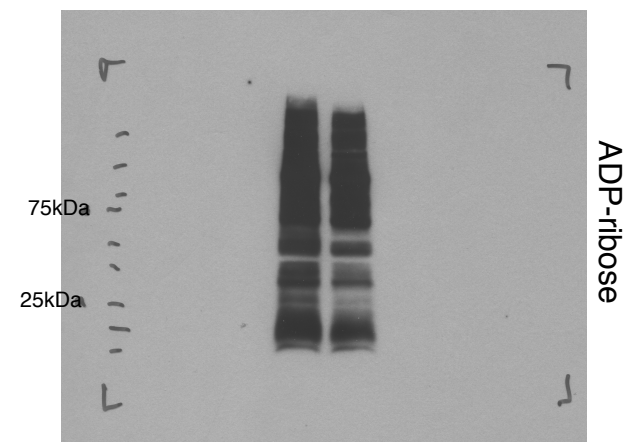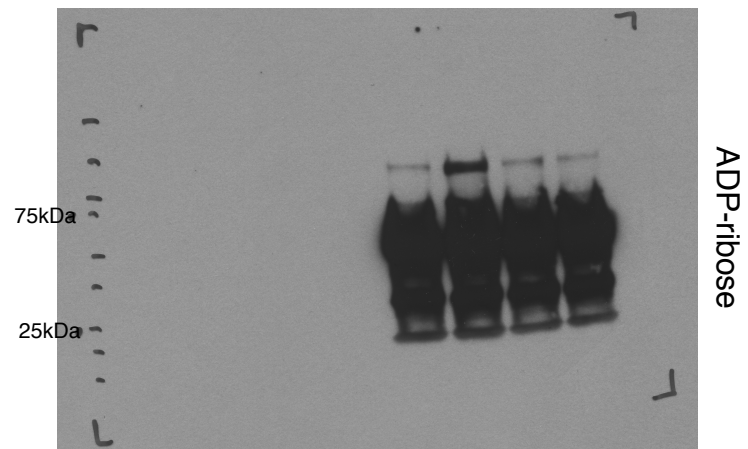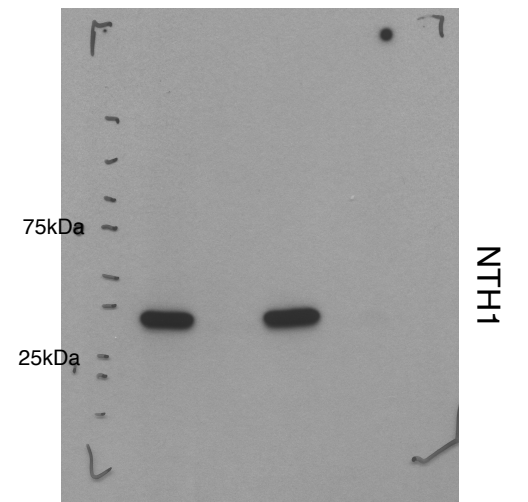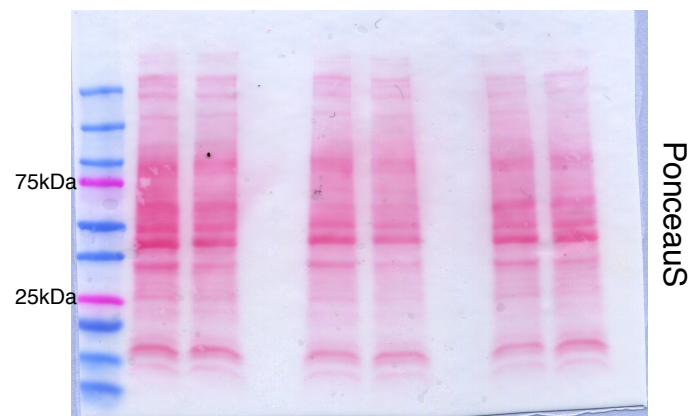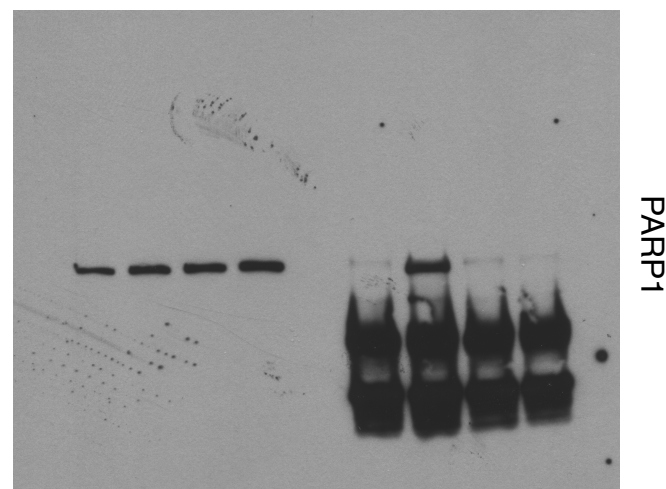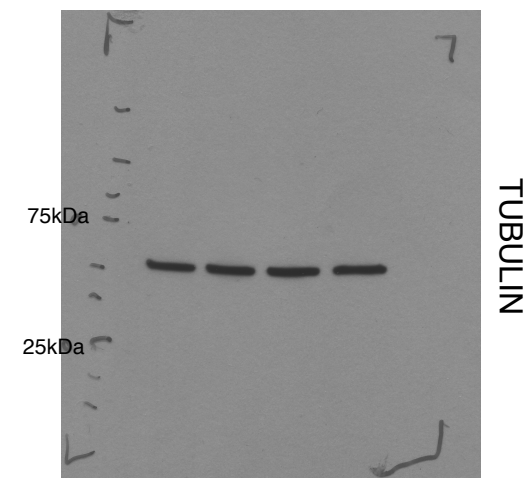

Extended Data Fig.6c

Extended Data Fig.6d

Extended Data Fig.6e

Supplement: Source Data Extended Data Fig. 6 — Unprocessed western blots. [file 41556_2021_792_MOESM25_ESM.pdf]

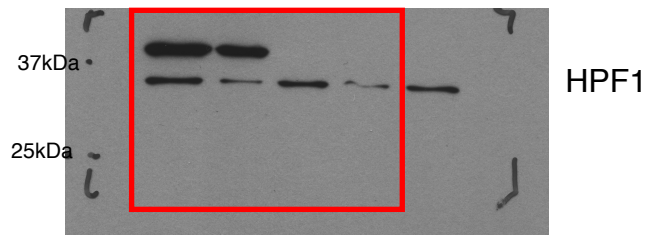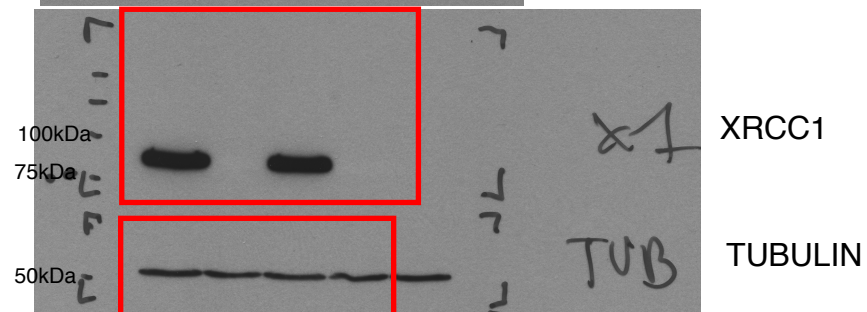

Extended Data Fig.7b

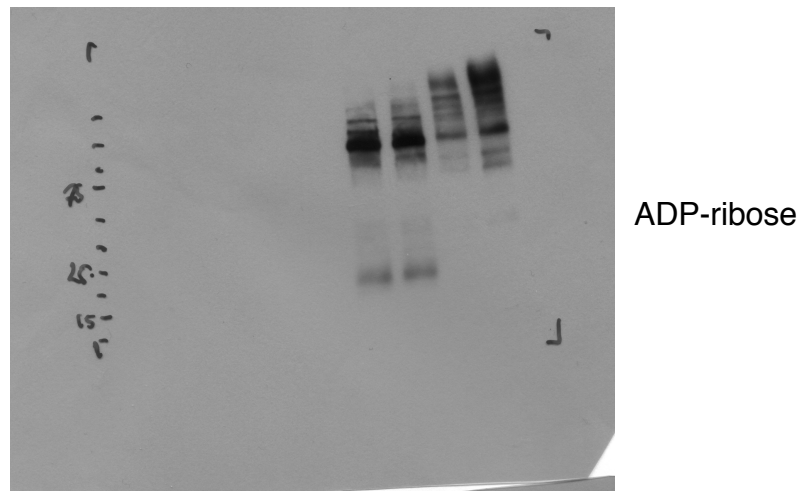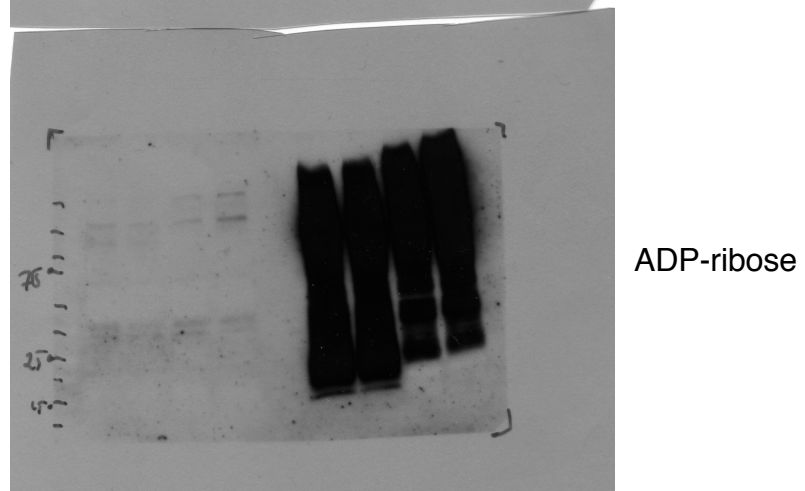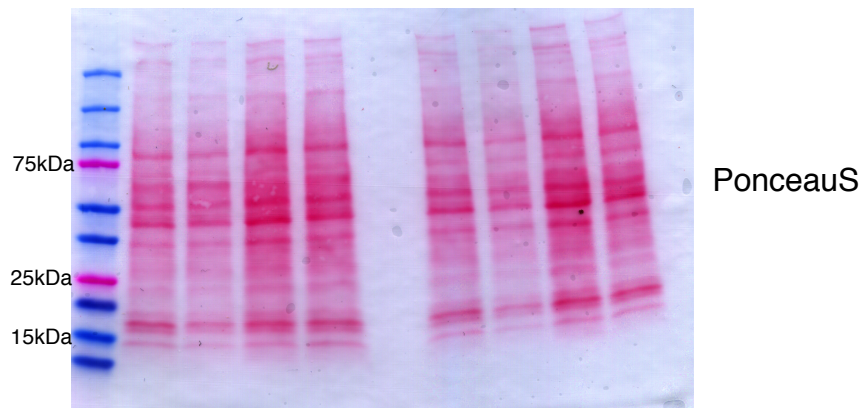

Extended Data Fig.7c

Supplement: Source Data Extended Data Fig. 7 — Unprocessed western blots. [file 41556_2021_792_MOESM27_ESM.pdf]

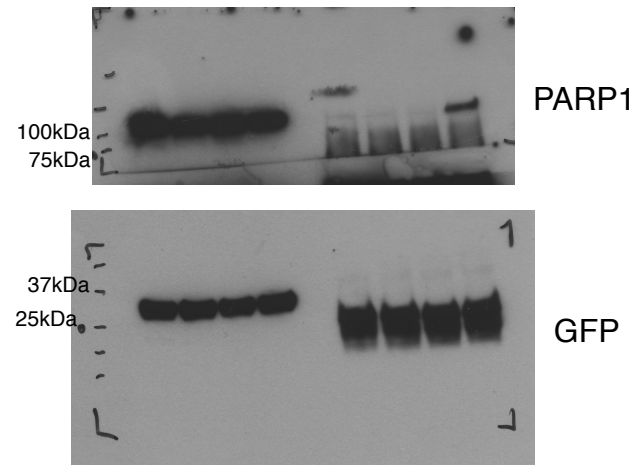

Extended Data Fig.10d

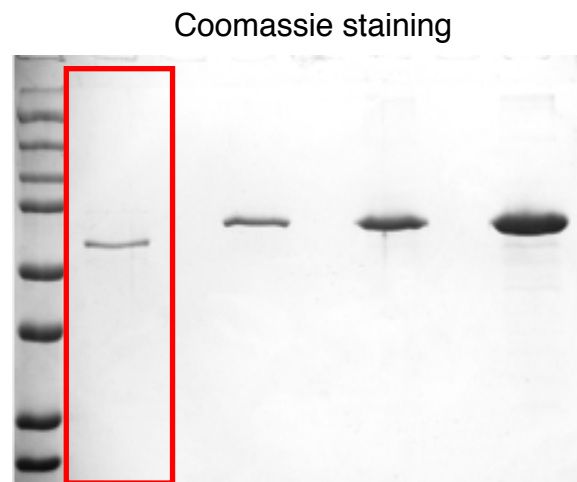

Extended Data Fig.10e

Supplement: Source Data Extended Data Fig. 10 — Unprocessed western blots. [file 41556_2021_792_MOESM31_ESM.pdf]
