# Supplementary material for: XRCC1 protects transcription from toxic PARP1 activity during DNA base excision repair
Source: Nat Cell Biol. 2021 Nov 22;23(12):1287–98. doi: 10.1038/s41556-021-00792-w (PMC8683375; doi:10.1038/s41556-021-00792-w)
Supplement: Source Data Extended Data Fig. 5 — Unprocessed western blots. [file 41556_2021_792_MOESM23_ESM.pdf]

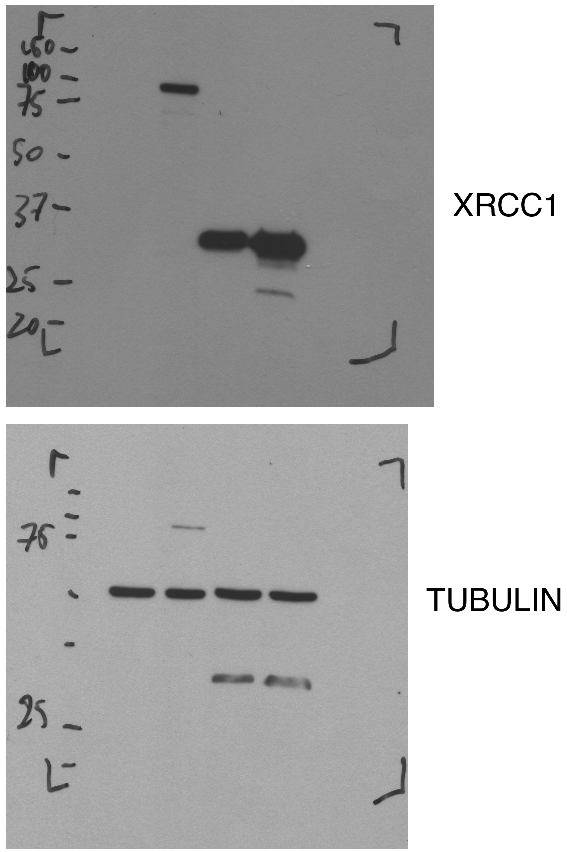

Extended Data Fig.5d

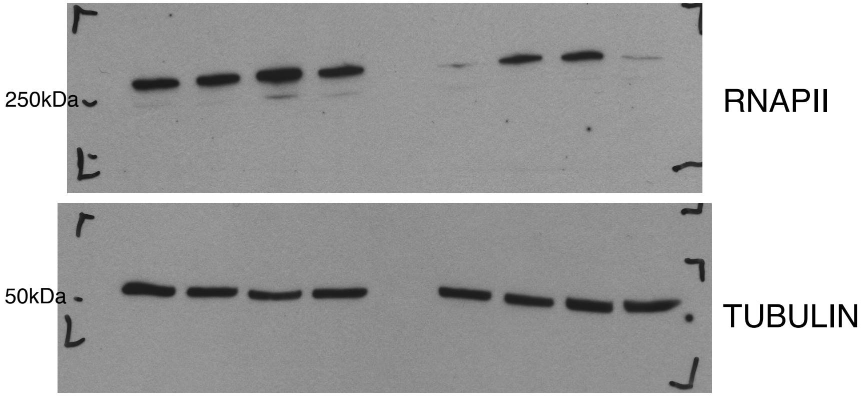

Extended Data Fig.5e

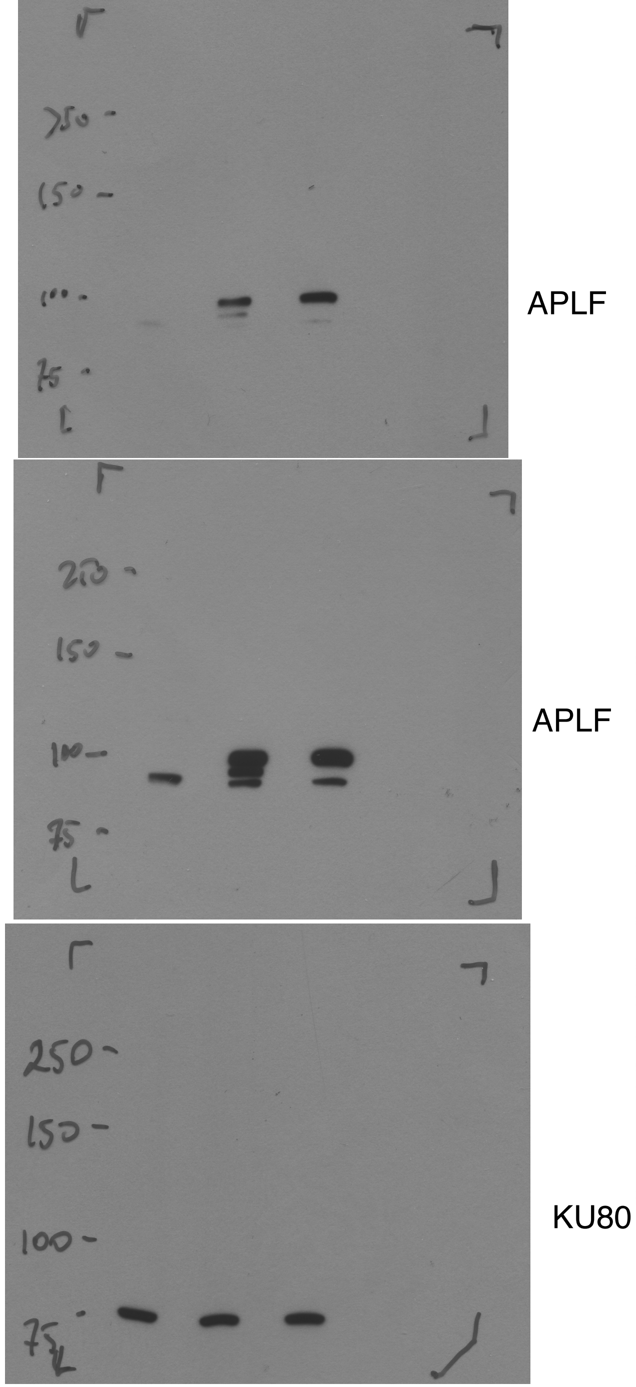

Extended Data Fig.5i

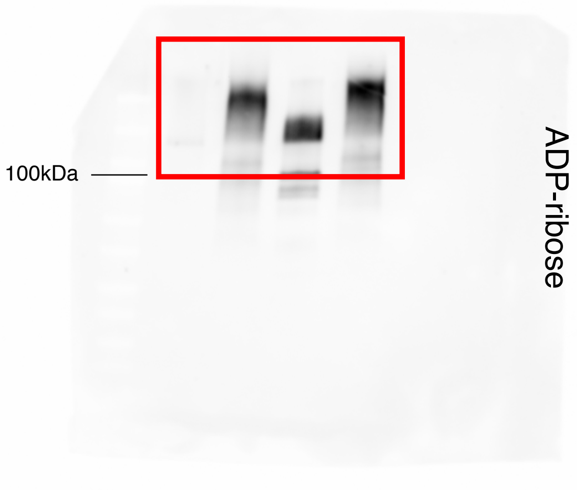

Extended Data Fig.5j
